# X-ray Absorption Fine Structure (XAFS) Studies of Oxide Glasses—A 45-Year Overview

**DOI:** 10.3390/ma11020204

**Published:** 2018-01-28

**Authors:** Valmor Roberto Mastelaro, Edgar Dutra Zanotto

**Affiliations:** 1 Physics Institute of São Carlos, University of São Paulo, 565-905 São Carlos, SP, Brazil; 2 Center for Research, Technology and Education in Vitreous Materials (CeRTEV), Federal University of São Carlos, 565-905 São Carlos, SP, Brazil; dedz@ufscar.br

**Keywords:** glass, structure, local-order, XAS, XANES, EXAFS, metal oxides

## Abstract

X-ray Absorption Fine Structure (XAFS) spectroscopy has been widely used to characterize the short-range order of glassy materials since the theoretical basis was established 45 years ago. Soon after the technique became accessible, mainly due to the existence of Synchrotron laboratories, a wide range of glassy materials was characterized. Silicate glasses have been the most studied because they are easy to prepare, they have commercial value and are similar to natural glasses, but borate, germanate, phosphate, tellurite and other less frequent oxide glasses have also been studied. In this manuscript, we review reported advances in the structural characterization of oxide-based glasses using this technique. A focus is on structural characterization of transition metal ions, especially Ti, Fe, and Ni, and their role in different properties of synthetic oxide-based glasses, as well as their important function in the formation of natural glasses and magmas, and in nucleation and crystallization. We also give some examples of XAFS applications for structural characterization of glasses submitted to high pressure, glasses used to store radioactive waste and medieval glasses. This updated, comprehensive review will likely serve as a useful guide to clarify the details of the short-range structure of oxide glasses.

## 1. Introduction

Structural studies of glassy materials have always been considered on two or three different length scales. Due to the absence of the (long-range) structural periodicity characteristic of crystalline materials, it is educative and necessary to define the local or short-range order and the medium-range order in glassy materials. A second aspect is to consider that these materials have some specific physical and chemical properties, which are not always observed in crystalline materials of the same composition. The challenge is then to establish a relationship between these properties and the short (1.5–3.0 Å) and medium-range (3.0–5.5 Å) orders [1].

The high relevance of structural studies of glasses is not only due to the interest in understanding the specific behavior of the physical and chemical properties of this class of materials but also due to the wide range of applications that are familiar to our daily life and their innumerable high-tech technological uses.

Very substantial progress in materials science was achieved using structural characterization adopting a wide range of X-ray diffraction techniques. Although this approach allowed the determination of the long-range order (LRO) atomic structure of single and poly crystals with high precision, in certain cases, due to the lack of LRO, the atomic structure cannot be adequately described. Among these cases, we can mention materials that only exhibit short or medium range orders, such as glassy and amorphous materials. Since local interactions determine, in many cases, the primary material properties, it is very important to characterize the local structure very accurately, using a local probe that does not depend on the existence of the long-range order. Different techniques have been adopted for the structural study and among them, the X-ray Absorption Fine Structure spectroscopy (XAFS) technique has been extensively used to characterize the short-range order because it is atomic species-selective and does not require the existence of the long-range order, thus allowing the structural characterization of crystal, glassy, liquid or even gaseous samples. Over the past four decades, the XAFS technique has undergone tremendous progress due to the availability of new generations of synchrotron light sources, advances in the theoretical description of the physical phenomena involved, and easy access to software packages, leading to a more accurate interpretation of short-range and medium-range orders and electronic structures (oxidation state) of different classes of materials.

**Figure 1:**
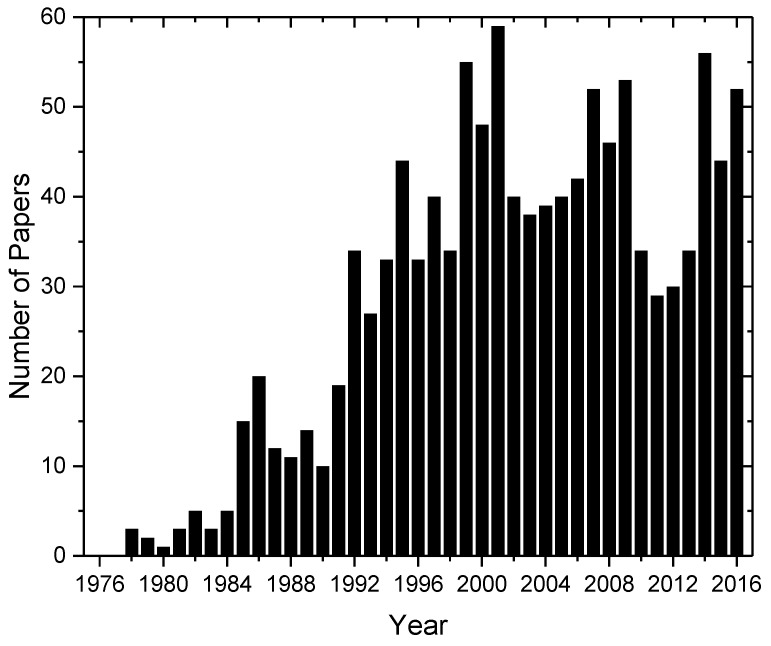
Evolution of the number of papers reporting on the structural characterization of glassy materials by XAFS spectroscopy. Source: ISI Web of Science.

In the past 45 years, XAFS has been widely used to characterize the short-range order and the electronic structures of different classes of glassy materials and also the medium-range order in some cases [2]. Figure 1 shows the number of published papers on experimental results of the structural characterization of glassy materials from 1975 to 2016. XAS (X-ray Absorption Spectroscopy), XAFS (X-ray Absorption Fine Structure), EXAFS (Extended X-ray Absorption Fine Structure), XANES (X-ray Absorption Near Edge Structure) acronyms together with the keywords “glass or glasses” were used as search keys in this bibliographic analysis in the ISI Web of Science. Figure 1 shows that the number of structural studies of glassy materials initially underwent a significant increase, then about 20 years ago, saturated at around 40–50 papers/year.

The glass types to which the XAFS technique has been applied include oxides, metallic, fluoride, chalcogenide and halide glasses. Also, besides the study of glassy materials under normal conditions, the XAFS technique has also been used to study the structure of synthetic or natural glasses, i.e., the study of glassy materials in their liquid state, as well as natural magma glasses.

In a pioneering paper published 10 years after establishing the physical fundamentals of XAFS, Greaves showed the importance of studying mineral glasses to better understand the structural properties of silicate melt magmas [3].

Two years later, Calas et al. selected some results in the literature about the study of the structure of glassy and mineral silicates to show the usefulness of XAFS in determining the short-range structure, in some cases, the middle range order of cations and anions in these materials [4]. In this paper, they present results from different authors on the structural characterization in glasses containing various types of glass forming cations, including Si, Al, Na, K, Ca, Ti, Fe, Yb and U. The authors also showed the great utility of XAFS for the study of vitreous samples containing a low concentration of some cations, such as yttrium and uranium.

Later, in 2002, Calas et al. published another paper describing the structure-property relationships in multicomponent oxide glasses using XAFS [1]. Among the most critical aspects of the structure-property relationship, they showed that the use of zinc as a stabilizer of the vitreous phase comes from its position in the network, where it acts as a network-forming element, implying in the presence of low-charge cations in its surroundings. Additionally, they investigated the structural role played by cations in oxide glasses that may occur in different types of coordination and their influence on the physical and chemical properties of these glasses [1]. The dependence between the colors of glasses and their composition when doped by transition elements was also investigated by these authors, taking as an example vitreous samples based on silicates and borates containing nickel. In this specific case, the authors observed that a wide range of colors observed in the samples could be explained by the fact that the nickel atom is present in different chemical environments, and is coordinated by 4, 5 or 6 oxygen atoms. Finally, these authors showed that the combination of information obtained by a wide range of X-ray spectroscopic and scattering methods, combined with numerical modeling, can provide an overview of the structural organization around cations present in glassy materials [1].

In a manuscript published in 2008, Dalba et al. presented two important examples of the application of the XAFS technique by measuring the EXAFS spectrum of several glassy systems [5]. The first example was the characterization of the local structure of rare earth ions dispersed in vitreous matrices or embedded in nano-crystallites nucleated in these same matrices after heat treatment [5]. Due to their disordered structure, it is difficult to obtain quantitative structural results of atoms located in outermost atomic layers, but these authors have shown that it is possible to determine the role of adding Al_2_O_3_, Na_2_O and TiO_2_ oxides to the SiO_2_ glass structure doped with Er. A significant result of their study is the non-observation of Er^3+^-Er^3+^ primary bonds, even at high arise from the existence of Er^3+^-Er^3+^ interactions over longer distances, which are not part of the capabilities to be observed in the EXAFS spectrum. In a second example, Dalba et al. described in detail studies of the local structure of silver atoms in binary borate glasses [5]. In this case, the authors emphasized the difficulty in describing the local structure of silver because it tended to establish a multi-coordination or to be present in different sites. In this case, unlike other vitreous systems, the correct interpretation of the local structure was obtained when the thermal disorder is also considered during the fitting procedure. The authors concluded that in the study of the structure of more complex vitreous materials and liquid systems, the XAFS technique complements characterization by X-ray diffraction and neutron techniques. While the XAFS technique is more sensitive to short-range order, diffuse scattering methods are more sensitive to structures at greater distances. In disordered systems, although thermal disorder effects can be added to the analysis, only the measurement of the EXAFS spectrum may be inadequate to correctly determine the local structure and, in this case, XANES measurements and the use of X-ray and neutron scattering techniques associated with theoretical calculations can give a correct view of the local structure, such as the average distance of ions in these complex materials.

In the present review, after a short description of the history of the XAS technique—where we describe the first time it was used for structural characterization of a glassy material—we will present a brief description of the physical phenomena involved with the technique, some experimental details and the basics about data analysis. Then, we will present some examples of applications of XAFS to study the structure of oxide-based glassy materials, mainly those containing Ti, Fe and Ni transition metals ions. We will show that by measuring the X-ray absorption spectra around the edge of a given atomic species (XANES spectrum), qualitative structural information can be obtained about the local and electronic structure and to associate it with the material properties. On the other hand, we will also present some examples where, to interpret more correctly the glass properties, it is necessary to measure and simulate the extended X-ray absorption spectrum (EXAFS spectrum). One focus of this review was the characterization of the role of Fe and Ti transition metal ions in different properties of vitreous oxides, as well as the essential role in natural glass and magma formation, nucleation and crystallization processes. At the end of the manuscript, we give some examples of XAFS applications for structural characterization of glasses submitted to high pressures, glasses used to store radioactive waste and medieval glasses.

## 2. A Short History of the XAFS Technique and Its Application to Glassy Materials 

When the energy of the incident X-ray photons increases, the absorption coefficient decreases continuously showing sharp increases each time, the photon energy is enough to excite an internal level of a given atom. These discontinuities in the absorption coefficient, *μ*(*E*), are called absorption edges. In the case of an isolated atom, *μ*(*E*) decreases monotonically after the absorption edge. If the atom belongs to a molecule or any particular structure, the variation of the absorption coefficient presents oscillations (also called fine structures) defined as EXAFS (Extended X-ray Absorption Fine Structure), which can extend from a few dozen to a few hundred electron-volts (eV) after the absorption edge. 

When we refer to the entire (before and after the edge) X-ray absorption spectra, the general preference is to use either XAS (X-ray Absorption Spectroscopy) or XAFS (X-ray Absorption Fine Structure). XANES (X-ray Absorption Near Edge Structure) referred to the absorption spectra near the edge (within ~30 eV) and EXAFS, referred to the extended part of the absorption spectra as described above. More details about the nomenclature used to describe the X-ray absorption technique can be found in references [6,7].

The presence of oscillations in the region after the absorption edge was first observed around 1920 [6,7], but its physical meaning that these oscillations are related to the structural order within short distances was only clarified around 1970 [8,9,10,11]. In that same period, the development of Synchrotron radiation laboratories allowed the realization of experiments that also helped to understand the evolved physical phenomenon and allowed for a more rapid and systematic study of different types of compounds presenting the structural characteristics described above. Over the past 45 years, very significant progress has taken place concerning the theoretical interpretation of physical phenomenon, the absorption edge (XANES) region [12,13,14] and in software.

**Figure 2:**
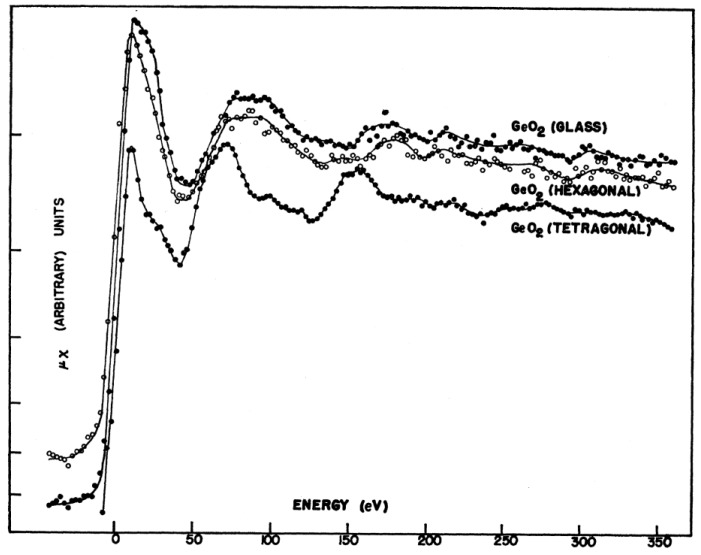
Ge K-edge X-ray absorption spectra of crystalline and vitreous GeO_2_ phases [15]. “Reproduced with permission from published by © American Physical Society.” (2017).

The first manuscript in which the phenomenon of X-ray absorption was used to study the structure of a glassy sample was published before establishing the physical interpretation of the XAFS phenomenon in a way that is currently accepted [15]. In that manuscript, only the absorption K-edge of germanium atoms in crystalline GeO_2_ (hexagonal and tetragonal) and glassy GeO_2_ were measured and compared [15]. Figure 2 shows the X-ray absorption spectra around the Ge K-edge of these three distinct materials showing a similarity between the absorption spectra of the vitreous and hexagonal GeO_2_ phases. According to the authors, this similarity was surprising and not expected from the theory used to describe the phenomena at that time.

The oscillations observed in the absorption coefficient (Figure 2) above the absorption edge were called EXAFS (extended x-ray absorption fine structure) by Prins and Lytle [7]. Another acronym has also been used to describe the phenomena after the absorption edge, XAFS (X-ray Absorption Fine Structure) comprehending the extended XAFS, referring to the EXAFS region, i.e., the fine structure well above an X-ray absorption edge and the fine structure close to an edge, i.e., the X-ray absorption near-edge structure (XANES) [7].

A fundamental advance in the development of X-ray absorption spectroscopy occurred at the beginning of the 1970s, when Sayers, Stern and Lytle applied the Fourier analysis to their point-scattering theory of X-ray absorption fine structure to invert experimental data formally into a radial structure function. Using this approach, they showed that it was possible to determine structural parameters, such as the distance from the absorbing atom and number of atoms and widths of coordination shells [8,9,10,11]. By analyzing the crystalline and vitreous germanium, they found that the first and second neighbors in amorphous Ge were equal to the crystalline distance within the accuracy of their experiment at that time. From these results, they concluded that the accuracy of the EXAFS technique was comparable to conventional X-ray or electron scattering methods, and that central feature of EXAFS was the ability to determine the near-neighbor surroundings about each (selected) type of atom in a complex material, since each atomic X-ray absorption edge occurs at a discrete and readily separable X-ray energy. 

With the interpretation of physical phenomena associated with X-ray absorption and with the possibility of collecting X-ray absorption spectra in synchrotron radiation laboratories, in the following years the XAFS technique was increasingly used to determine the local structures of different families of glassy materials, for example, metallic glasses [16,17], lithium germanate glasses [18], sodium silicate glasses containing iron [19], vanadium iron phosphate glasses [20], chalcogenide glasses [21], barium fluorozirconate glasses [22] and silver borate glasses [23]. 

## 3. Physical Principle of XAFS

Some excellent textbooks and reviews on X-ray absorption spectroscopy have been published. Therefore, we only present here some essential principles and theoretical elements evolving the XAFS technique [12,24,25,26,27,28,29].

**Figure 3:**
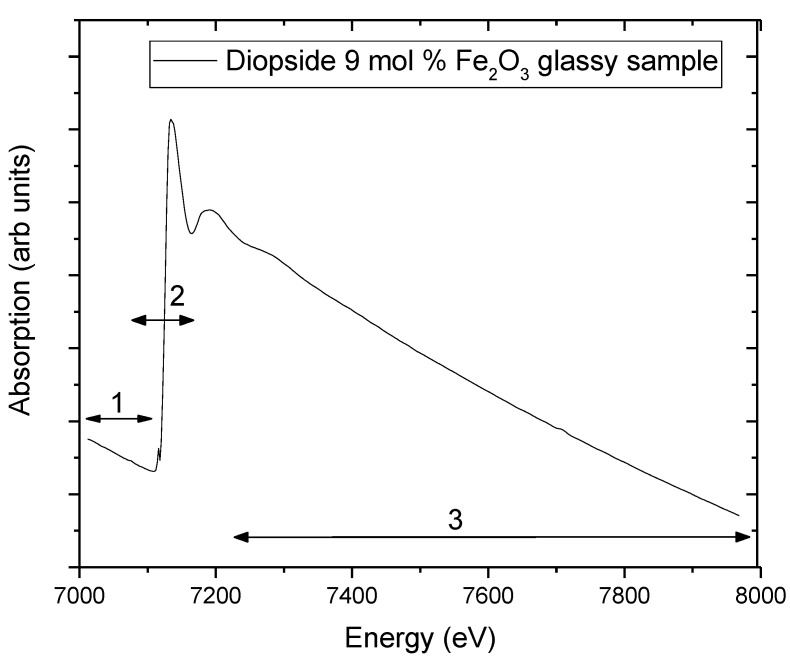
X-ray absorption spectra collected at the iron K-edge of the CaMgSi_2_O_6_-9 mol % of Fe_2_O_3_ Sample. 1: pre-edge region; 2: Near edge region; 3: extended X-ray absorption region. (Mastelaro et al. unpublished).

As we mentioned before, the XAFS spectrum results from the absorption coefficient, *μ*(*E*), as a function of photon energy above the absorption edge. Figure 3 shows a typical absorption spectrum collected at the Fe K-edge of an iron-doped glassy diopside sample (CaMgSi_2_O_6_-9 mol % of Fe_2_O_3_).

The most common and simple way of measuring the absorption coefficient, *μ*, as a function of energy is using the transmission mode, i.e., with the X-rays passing through the sample. In this case, *μ* or *μx*, where *x* is the thickness of the sample, can be written as:

(1) $$\mu x=\ln\frac{I}{I_{0}},$$

where *I*_0_ and *I* are respectively the intensity of the incident and a transmitted beam of photons. The coefficient *μ* depends both on material properties and photon energy (*hν*). If the absorption coefficient is plotted as a function of the energy as shown in Figure 3, the experimental data show three general features: (1) the absorption coefficient decreases gradually with increasing energy; (2) the presence of a sharp rise at specific energies called edge, which roughly resembles step-function increases in the absorption spectra; and (3) above the edge, a series of oscillatory structures that modulate the absorption. The second feature, or the edge, occurs when the photon energy corresponds to a threshold (*E*_0_) for a core electron excitation. The edge position is unique to a given absorption atom since it corresponds to the binding energy of the photoelectron.

In the X-ray absorption spectrum analysis shown in Figure 3, three distinct regions are typically highlighted and analyzed regarding the physical processes involved in each one:

Region 1: near or before the absorption edge may have absorption peaks due to excitation of electrons from the ground state to bound states. From the transitions observed in the pre-edge region, information can be obtained about the site symmetry of the absorbing atom via comparative analysis with the absorption spectra of standard samples.

Region 2: this region is called XANES (X-ray Absorption Near Edge Structure), where effects of multiple diffusion and multi-electronic interactions of the photoelectron occur. A comparative analysis of the edge position in energy with standard samples determines the oxidation state of the absorbing atom.

Region 3: EXAFS region from approximately 50 to 1000 eV after the absorption edge, where events of simple scattering predominate, thus obtaining information such as the coordination number, the interatomic distance and the thermal/structural disorder around the absorber atom.

We should emphasize that this division of the X-ray absorption spectrum into three regions is purely conditional and the interval of each region may vary for different compounds. Therefore, the term X-ray absorption fine structure (XAFS), which corresponds to the complete absorption spectrum, is often used in the modern literature for the entire oscillating component beyond the absorption edge [24].

To understand and model the XANES region of the spectrum, sophisticated calculations should be used taking into account effects of multiple scattering [12]. On the other hand, EXAFS oscillations, dominated by single scattering, can be interpreted by a relatively simple mathematical treatment. The availability of reliable and simplified data processing techniques has transformed EXAFS into a widely used as a structural characterization technique.

### 3.1. The EXAFS Region

From a qualitative point of view, the probability that an X-ray photon is absorbed by an electron located at the core level depends on the initial state and final state of the electron. The initial state is located in a ground state level. The final state corresponds to the photoelectron ejection and can be represented by an outgoing spherical wave, originating at the absorbing atom. If the absorber atom has other atoms around it, the emitted wave representing the photoelectron will be backscattered by these neighboring atoms, producing a backscattered wave. The final state is then defined as the sum of the wave emitted and the backscattered wave by each of the neighboring atoms. The process of interference of these waves is the origin of the sinusoidal variation of the absorption coefficient *μ*, defined as EXAFS.

The amplitude and frequency of sinusoidal modulation of *μ*(*E*) depend respectively on the number and type of neighboring atoms and their distances from the absorber atom. This simple description of the physical phenomenon associated with the EXAFS oscillations was formulated in a general theory that presupposes the existence of simple backscatter processes between the absorber atom and the first neighboring atoms, shorter photoelectron wavelengths, a localized spherical wave and the study of the structure within a short-range distance [24,25,26,27,28,29].

The modulation of the absorption coefficient, normalized by the background absorption, *μ*_0_, can be written as:

(2) $$\chi (E)=\frac{\mu (E)-\mu_{0}(E)}{\Delta \mu_{0}\left(E\right)},$$

where *μ*(*E*) is the measured absorption coefficient, *μ*_0_(*E*) is a smooth background function representing the absorption of an isolated atom, and ∆*μ*_0_ is the measured jump in the absorption *μ*(*E*) at the threshold energy *E*_0_.

The wave vector *k* of the ejected photoelectron due to absorption of a photon of X-rays can be described as

(3) $$k=\frac{\sqrt{2m\left(E-E_{0}\right)}}{\hbar^{2}},$$

where *E* is the energy of the incident photon and *E*_0_ is the energy of a particular absorption edge.

In the case of energies 50 eV above the absorption edge and the case of systems with the thermal and static disorder, the EXAFS oscillations, *χ(k*), can be described as [10,28]:

(4) $$\chi (k)=-S_{0}^{2}\underset{j}{\sum }\frac{N_{j}}{kR_{j}^{2}}\;|f_{j}\left(\pi ,k\right)|e^{-2\sigma^{2}k^{2}}e^{\frac{-2R_{j}}{\lambda \left(k\right)}}\;sin\left(2kR_{j}+2\delta_{j}\left(k\right)\right),$$

where *f_j_* is the backscattering amplitude of each neighbor *N_j_* atom type *j*. This equation contains terms corresponding to a damping disorders such as Gaussian with a Debye-Waller factor *σ*^2^, and the term *exp*(−2*R_j_/λ(k*)) related to inelastic losses in the diffusion process where *λ*(*k*) is the mean free path of the ejected electron, and *S*_0_(*k*) is the reduction term due to multi-electron effects. Finally, the equation also contains the term dependence with the inverse of the distance between the atoms, 1/*kR_j_*^2^, and the term sine interference that also depends on the distance between the atoms (2*kR_j_*) and phase difference *δ_j_*(*k*). Factor 2 results from the fact that the photoelectron travels a path back and forth between the absorber atom and the neighboring atom, the backscattered wave is 2*kR_j_* about the wave emitted.

The structural parameters involved in the EXAFS equation are coordination number (*N*), the interatomic distance (*R*) and the Debye-Waller factor (*σ*), which includes two contributions, one dynamic originating from atomic vibrations and another one, which is static, originating from structural disorder within a given coordination sphere. 

Once it was assumed that *χ*(*k*) can be represented by a linear combination of sine waves of each layer of coordination, in principle, each contribution can be separated by applying a Fourier transform. 

By obtaining and analyzing the EXAFS signal structural information from a particular layer can be obtained, such as the number of neighbors (*N_j_*), the average distance between the absorbing atom and its first neighbors (*R_j_*) and the factor of thermal disorder and static (*σ_j_*). For this, it is necessary to know a priori the electronic parameters containing the EXAFS equation: *λ*(*k*), *f*(*k*), *δ*(*k*) and *S*_0_^2^(*k*). For these electronic parameters, currently, the most used method is a theoretical calculation using programs such as the FEFF code [12,29]. It is important to emphasize that the FEFF code before the FEFF9 version estimated the Debye-Waller factors using a semi-empirical Einstein model or the Debye vibrational density of states (VDOS) model [30]. It was shown that these models are not efficient to calculate these factors in materials having a higher degree of thermal and structural disorder like glasses, and other approaches have been proposed [30]. For example, Dimakis and Bunker used an *ab initio* quantum chemistry approach including density functional theory and semi-empirical approaches to calculate single and multiple Debye-Waller factors parameters in a variety of materials [31]. Alternatively, the equation of motion technique was used by Poiarkova and Rehr to calculate these parameters [32]. In FEFF9 code, these limitations were avoided by calculating the VDOS using a continued fraction representation of the lattice dynamical Green’s function (LDGF) generated with the iterative Lanczos algorithm [30].

### 3.2. The XANES Region

The definition of the energy range that separates the XANES region, where the phenomena of multiple scattering are predominant, from the region of EXAFS where single scattering is predominant, is somewhat arbitrary. In the region of the XANES spectrum, the ejected photoelectron kinetic energy is weak and, therefore the mean free path is relatively significant. The contributions of neighboring atoms located at longer distances are more important than the EXAFS region and, the wave associated with the photoelectron can give the origin of multiple scattering. 

Recently, substantial progress has been made in developing programs that perform ab-initio calculations of EXAFS spectra, but mostly, for the analysis of XANES spectra [24,29]. When we analyze the XANES spectrum of glassy samples, their study is made using a comparison with standard (crystalline) samples to obtain information about the site symmetry and oxidation state of the absorber atom [29].

### 3.3. Measurement of the X-ray Absorption Spectra

Different methods have been developed to measure the X-ray absorption spectrum, and each one has its advantages and disadvantages. The most straightforward method that has often been used to study glassy materials is the transmission mode, where the intensity of the incident (*I*_0_) and transmitted photons (*I*) by a sample of thickness x is measured simultaneously. The transmission method is suitable for studying samples where the concentration of the studied atom is relatively high, and the sample thickness can be very low. Measurements in transmission mode are susceptible to sample quality regarding their homogeneity, which, in particular, has a very important influence on the amplitude of EXAFS oscillations. The measurement of the EXAFS or XANES spectra using this mode requires that the sample present a constant thickness and does not contain holes that may cause the x-ray beam to pass directly without reaching the sample.

The absorption spectrum of X-rays can also be measured by two other methods, an indirect way, the fluorescence mode, and the total electron yield mode. For dilute systems, a fluorescence method was used to enhance experimentally the relatively weak EXAFS signal from the bulk absorption background of the host matrix. This detection scheme utilizes the fact that an inner shell vacancy may relax by undergoing a radiative transition from a higher energy occupied shell. If the element to be studied is of the order of 1% of the sample in a matrix where the atomic number of the elements is slightly different, the only way to obtain an adequate absorption spectrum concerning signal to noise ratio is by way of fluorescence. If the element in the sample is at a high concentration and the sample is deposited on a substrate, the most suitable detection method to measure the absorption spectra is the conversion of electrons or Total Electron Yield.

### 3.4. EXAFS and XANES Data Analysis

We will focus on the method of obtaining and analyzing the EXAFS spectrum, as that is how quantitative information on the short-range order structure can be obtained. As previously mentioned, in the case of glassy materials, due to its amorphous characteristic, the analysis of the XANES spectrum is made only by comparing the sample with standard compounds, where the objective is to check the local symmetry aspects and oxidation state of the atom being analyzed. For this comparison, XANES spectra are normalized to interpret variations in the intensity.

### 3.5. Extraction of the χ(k) EXAFS Signal

To obtain the structural parameters from the EXAFS region of the X-ray absorption spectrum, a procedure was established that consists of converting the raw absorption data into normalized EXAFS oscillations *χ*(*k*) and then extracting the structural parameters. However, depending on the complexity of the studied system, each stage of treatment should be developed very carefully.

**Figure 4:**
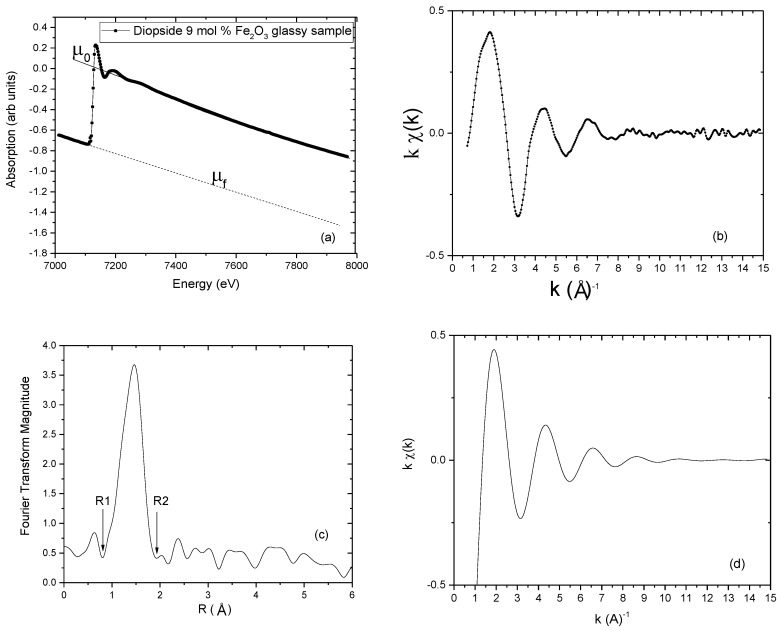
Data extraction process for analysis of EXAFS of CaMgSi_2_O_6_-9 mol % Fe_2_O_3_ glassy sample: (**a**) pre-edge background subtraction and normalization; (**b**) EXAFS oscillations; (**c**) Fourier transform magnitude and (**d**) EXAFS inverse Fourier transform spectra related to the first coordination shell. (Mastelaro et al., unpublished).

As the absorption coefficient is obtained as a function of the X-ray photon energy, initially we should obtain the *χ*(*E*) function from the atomic absorption *μ*_0_(*E*). The first step is to remove the background absorption *μ_f_*(*E*) using a polynomial function, which is extrapolated to the region after the absorption edge (Figure 4a).

Then, in the region after the absorption edge, we calculate the monotonous decay of the absorption coefficient *μ*_0_(*E*) using a polynomial function generally of a degree between 3 and 6. The EXAFS modulations are then obtained after the subtraction of background absorption and its normalization (Equation (2)). The calculation of the function *χ*(*k*) from function *χ*(*E*) can be obtained through Equation (3).

The determination of the atomic absorption is the most delicate step during the procedure of extraction of the EXAFS spectrum. A miscalculation of the polynomials can lead to *χ*(*k*) signal with the distorted signal and truncated abnormal at a low frequency. The spectrum *χ*(*k*) is presented as a damped sinusoid so that for large values of *k*, the signal is practically zero. To better visualize the spectrum at *k* values, *χ*(*k*) spectrum is multiplied by *k^n^*, and n is equal to 3.

A check of the quality with which the EXAFS signal has been extracted is by verifying that the oscillations are symmetrically distributed around zero. After this procedure, the EXAFS oscillations shown in Figure 4b can be obtained.

### 3.6. The Fourier Transform

Lytle, Stern and Sayers were the first to show that to separate the different contributions from individual coordination shells we can Fourier transform (FT) of *χ*(*k*) into a distance (real) space [8,9,10]. They showed that the FT modulus showed peaks situated at distances comparable to those obtained by X-ray diffraction. The Fourier transform of the *χ*(*k*) EXAFS spectra in a *k* region defined between *k_min_* and *k_max_* gives origin to a modified radial distribution function, *ρn*(*r’*), which provides a qualitative interpretation of the local structure around the absorber atom (Figure 4c). Multiplication by a *k^n^* factor is used to compensate the sharp decrease of the *f*(*k*) amplitude function as *k* increases. The window parameter defines the utile integration zone of *χ*(*k*) spectra and the window choice influences the FT peak form and resolution and should then be the same when a set of samples is compared. The Fourier transform aims to separate the different contributions of the coordination spheres around the absorber atom. To select a specific coordination sphere of coordination we want to analyze separately, we used a window between R1 and R2 (Figure 4c) and calculated an inverse Fourier transform of the region encompassed by the window (Figure 4d). Usually, for glassy samples, the Fourier transform consists of the main peak due to the structural characteristics of glassy samples, only a short-range order structure comparable to its crystalline phase. In addition to the first coordination sphere, further shells located at higher distances presents low-intensity peaks due to several factors such as the complexity of the sample composition (presence of different types of atoms). Additionally, a high level of structural disorder gives origin to destructive interference effects that cause a significant decrease in peak intensities at higher distances.

### 3.7. Fitting Procedure: Determination of Structural Parameters

The fitting procedure is the last step of the analysis of the EXAFS spectrum. This step leads to quantitative information of the short-range order structure (*N*, the first neighbor number situated in a determined shell, the mean bond-length between the absorber, *R*, and their first neighbors and *σ*, the Debye-Waller factor) through the theoretical fit of the experimental EXAFS spectrum. 

Among the different software packages, which are available for this fitting procedure, we can highlight the WINXAS [33], ATHENA and ARTEMIS (actually DEMETER) [34] and MAX: Multiplatform Applications for XAFS [35]. As was described before, to calculate the theoretical spectrum, it is necessary to provide atomic parameters (*λ*(*k*), *f*(*k*), *δ*(*k*) and *S*_0_^2^(*k*)) presented in the EXAFS equation. These atomic parameters can be adequately calculated from the FEFF9 program [24,29,30].

## 4. Examples of Using the XAFS Technique to Study the Atomic and Electronic Structure of Glassy Oxide Materials

### 4.1. XAFS Studies of Ti and Fe Transition Elements in Oxide Glass Materials

Oxide glasses containing transition metal oxides are considered relevant because they exhibit interesting spectroscopic and electrical properties and can be used in solid-state lasers, phosphors, solar energy converters, plasma display panels, as well as electronic and optical devices [13,36,37]. It has been shown that structural and electronic properties of these glasses, as well as their optical, magnetic and other properties, depending on the short-range order symmetry and different valence states of the transition metal ions present in the glass matrix.

The study of the chemical environment of transition elements, such as Ti, V, Co, Fe, Ni in glassy oxide materials has attracted the attention of many researchers since XAFS became available, and proved to be a handy tool in the study of the role of these ions [20,38,39,40,41,42,43,44]. Although the XAFS technique has been used to study the coordination of different transition elements inserted in glasses, titanium and iron have been the most studied by XAFS. 

Different works have shown the importance of the knowledge of the titanium coordination in oxide glasses and melts because its presence can affect several physical properties, including color, thermal expansion, compressibility, elastic constants, sound velocity, densities, viscosities and nucleation rates [38,45,46,47].

The pre-edge features of the Ti K-edge XANES spectrum located before the absorption edge are commonly attributed to transitions from *1s* energy levels of Ti to the Ti_3d_/O_2p_ molecular orbital’s [48,49,50,51]. The *3d* transition is forbidden by dipole selection rules but is allowed when *p-d* orbital mixing occurs, such as when Ti is located in a TiO_4_ tetrahedron or a (^[5]^TiO)O_4_ site, i.e., without a center of symmetry. The height and position of the pre-edge feature are direct functions of the degree of *p-d* mixing, site distortion, oxidation state and experimental resolution [48,49,50,51].

The first XAFS studies of short-range order symmetry of Ti atoms in TiO_2_-SiO_2_ glasses were developed by Gregor et al. [38] and Sandstrom et al. [39]. In these studies, using XANES and EXAFS data, the authors showed that the coordination of Ti in TiO_2_-SiO_2_ glasses depends on the amount of TiO_2_, and is predominantly coordinated by four oxygens for higher concentrations of TiO_2_, which supported previous studies regarding the calculation of molar refractivity of these glasses.

**Figure 5:**
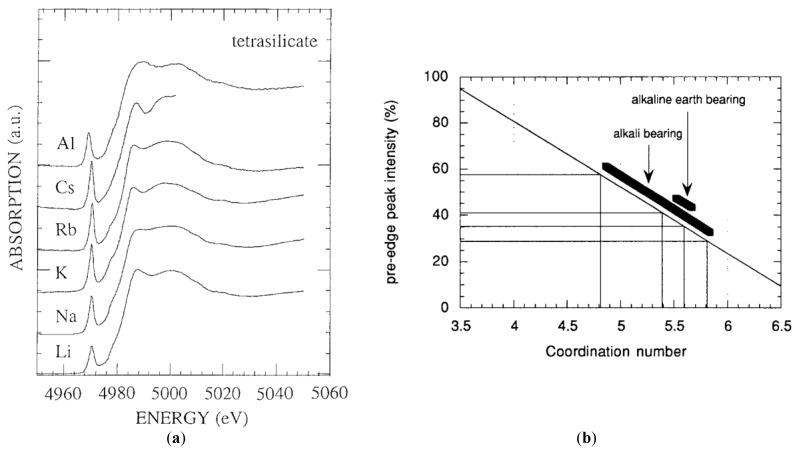
(**a**) Ti K-edge XANES spectra for alkali-bearing glasses of tetrasilicate composition and (**b**) the derivation of the average coordination number for different glasses based on the regression curve obtained from the analysis of XANES spectra of minerals compounds [47]. “Reproduced with permission from published by © Springer.” (2017).

In 1994, Dingwell and co-authors presented a more detailed study about the coordination of Ti atoms in Li, Na, K, Rb, Cs, Ca, Sr and Ba metasilicate and tetrasilicate glasses, as well as in Al_2_O_3_-TiO_2_-4SiO_2_ glass [47]. These authors were the first to present a direct correlation between the mean coordination number (CN) of the titanium atoms and the pre-edge peak intensity present before the absorption edge of the XANES spectrum of titanium. For this study, they measured and analyzed the relationship between the pre-edge peak intensity in a series of crystalline mineral samples (oxides, titanates, and silicates), where the coordination of the titanium atom by oxygen atoms was well known. Thus, assuming the correlation between the pre-edge peak intensity and the mean coordinate number around the titanium atom, they were able to apply this result to the analysis of the vitreous samples, thus obtaining more quantitative structural information. Figure 5a shows, as an example, the XANES spectra of a series of samples of alkali-bearing glasses of tetrasilicate composition where the variation of the intensity of the pre-edge peak can be observed depending on the composition of the sample. Figure 5b shows that the relationship between the coordination number of the titanium atoms and the intensity in mineral samples is linear. Regarding the vitreous samples analyzed in this work, the authors observed that the data obtained through linear regression are CN between 5.4 and 5.6 for the alkaline-earth silicate glasses and between 4.8 and 5.8 for the alkaline silicate glasses, respectively. The authors concluded that the average coordination of the titanium atoms was directly related to the dependence of specific physical properties of these glasses such as density, thermal capacity, and compressibility with their composition.

**Figure 6:**
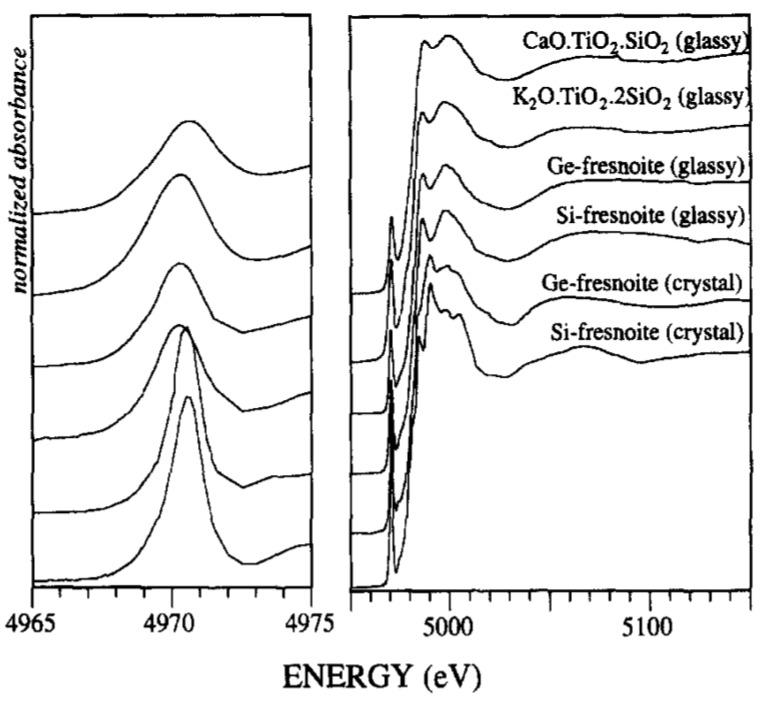
Ti K-edge XANES spectra of crystalline and glassy silicate and germanate fresnoite in comparison with calcium titanium metasilicate and potassium titanium disilicate glasses. **Left**: detail of the normalized pre-edge peak; **right**: normalized XANES spectra [48]. “Reproduced with permission from published by © Elsevier.” (2017).

A better understanding of the short-range order and electronic structure of titanium atoms in a wide range of synthetic and natural glassy systems, under ambient conditions as well as melting temperatures and high pressures, was provided by Farges et al. [2,45,48,52,53,54,55]. One of the significant interests of Farges and co-authors’ papers was to correlate (by a direct structural study) the anomalous high variation in heat capacities in Na and K-bearing titanosilicate melts at temperatures just above the glass transition temperature (T_g_) that were measured by different authors [56,57]. Since their first paper published in 1996, Farges et al. have used the strategy of correlating the normalized intensity, position, FWHM and area of Ti K-edge pre-edge feature with the coordination number of the titanium atoms in a wide range of model crystalline oxide compounds containing titanium in coordination ^[4]^Ti, ^[5]^Ti and ^[6]^Ti. This correlation was then used to study the coordination of Ti atoms in disordered materials such as glasses at room and melting temperatures [47,48,49,50,51,52,53]. Using this strategy, Farges et al. initially studied glassy fresnoite [48]. Figure 6 shows the XANES spectrum and in more detail, the pre-edge region of Si and Ge-based fresnoite glassy samples in comparison with their crystalline phases, as well as a calcium titanium metasilicate and potassium titanium disilicate glass compositions [48]. By comparing the normalized height and position of pre-edge peaks of fresnoite glasses with those obtained from model oxide crystalline compounds, they verified that although crystalline fresnoite presents only ^[5]^Ti coordination, glassy fresnoite samples presents ^[5]^Ti (60%) predominantly, but also ^[4]^Ti and ^[6]^Ti coordination. Additionally, Farges et al., using ab-initio calculation, determined the XANES spectra of oxide crystalline materials and glasses based on the structural information obtained from pre-edge peak analysis [48]. They found that the coordination square pyramidal geometry is the most plausible model for the short-range order structure around Ti in glassy fresnoite [48]. 

Later, in a series of four high-quality papers, Farges and co-authors presented a detailed studied about the short-range order structure of Ti^4+^ in selected oxide model compounds and Na, K, and Ca-bearing titanosilicate glasses and melts [2,45,52,55]. One of their main aims in these papers was to test the hypothesis proposed by different authors that the changes in the Ti coordination number in these titanosilicate melts were the possible structural explanation for the anomalous behavior observed in the heat capacities of these glasses [56,57]. In one of these papers [45], Farges et al. also showed essential aspects related to the data collection of XANES spectra concerning the energy resolution, which they consider an important factor when the XANES spectra of some transitions elements, such as Ti is collected because the pre-edge features could be quite narrow, around 1 eV wide. By testing different monochromators and different slits opening on the energy resolution at the Ti K-edge, they concluded that the energy resolution should be adequate to resolve the pre-edge features and that the comparison of pre-edge features collected at different experimental conditions should then be avoided [45]. 

**Figure 7:**
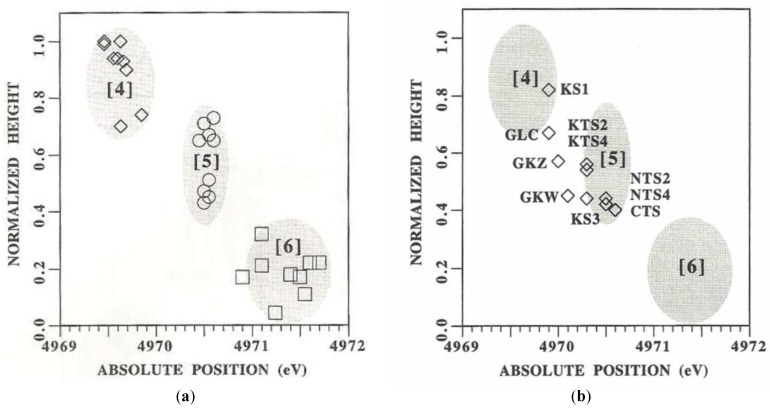
(**a**) Normalized pre-edge height versus energy position for Ti K-edge pre-edge peaks for a series of Ti crystalline model compounds (see Table I in Reference [2] for more details) [2]; (**b**) Normalized height versus energy position for the pre-edge peaks in Na, K and Ca titanosilicate glasses compared with the three Ti coordination number domains [2]. (KS1 = 58.35SiO_2_-0.26Al_2_O_3_-38.67K_2_O-2.72TiO_2_; KS3 = 64.67SiO_2_-0.27Al_2_O_3_-32.35K_2_O-2.71TiO_2; _KTS2 = 40.3SiO_2_-31.7K_2_O-0.02Na_2_O-26.6TiO_2_; KTS4 = 46.6SiO_2_-37.6K_2_O-16.0TiO_2_; NTS2 = 46.1SiO_2_-0.07K_2_O-24.0Na_2_O-30.6TiO_2_; NTS4 = 49.3SiO_2_-35.0Na_2_O-15.7TiO_2_; GLC = 67.27SiO_2_-11.67Al_2_O_3_-15.25K_2_O-5.81TiO_2_; GKZ = 69.30SiO_2_-11.39Al_2_O_3_-4.95CaO-8.23K_2_O-6.13TiO_2_; GKW = 71.70SiO_2_-11.92Al_2_O_3_-10.11CaO-6.27TiO_2_; CTS = 33.33SiO_2_-33.33CaO-8.23K_2_O-6.13TiO_2_. “Reproduced with permission from published by © Elsevier.” (2017).

From the analysis of about 27 different titanium-based oxide model compounds of coordination four (^[4]^Ti), five (^[5]^Ti) and six (^[6]^Ti), Farges at al. constructed a graph correlating the energy position of the XANES pre-edge peak and its normalized height so as to identify three domains for this coordination as shown in Figure 7a [2]. By the analysis of only the pre-edge height alone, they showed that it is not possible to distinguish between ^[5]^Ti and a mixture of 50:50 of ^[4]^Ti and ^[6]^Ti, therefore the pre-edge peak energy should only be considered. After identifying these coordination domains of Ti-based model compounds, they measured the height and energy positions of a series of Ti-bearing glasses, and they observed (Figure 7b) that these glasses present significant amounts of ^[5]^Ti and ^[4]^Ti coordination [2]. 

Additionally, based on the results of pre-edge peak position and height obtained from XANES spectra of Ti-bearing glasses, Farges at al. also analyzed the role of Ti as both network former and modifier, the influence of Ti content and the cation substitution on Ti coordination [2]. They also showed that the intensity of the main edge relative to the edge jump after proper normalization appears to be a direct function of the degree of short-range ordering around Ti, making XANES a sensitive probe of medium-range order structure around a cation in the studied glasses [2,45]. Based on the experimental data, bond valence models and ab-initio multiple scattering calculations, they showed that is a good strategy for probing the medium-range order structure around Ti atoms and that these results could be extended to other cations [2].

Farges et al. analyzed the structural behavior of Ti atoms as a function of the temperature in five different glassy systems by analyzing XANES and EXAFS spectra [52]. They concluded that no evidence was found for significant changes in the first-neighbor coordination shell of Ti in the studied glasses for temperatures just above T_g_, where the heat-capacity anomalies were observed [46]. Based on their results, they suggested that a possible cause of the heat capacity anomalies in the Na and K-titanosilicate glasses involves major changes in the configurations of Na and K in percolation domains where titanyl bonds of (TiO)O_4_ moieties are concentrated at the boundaries between alkali-rich and network former rich areas of the glass/melt structure [52].

**Figure 8:**
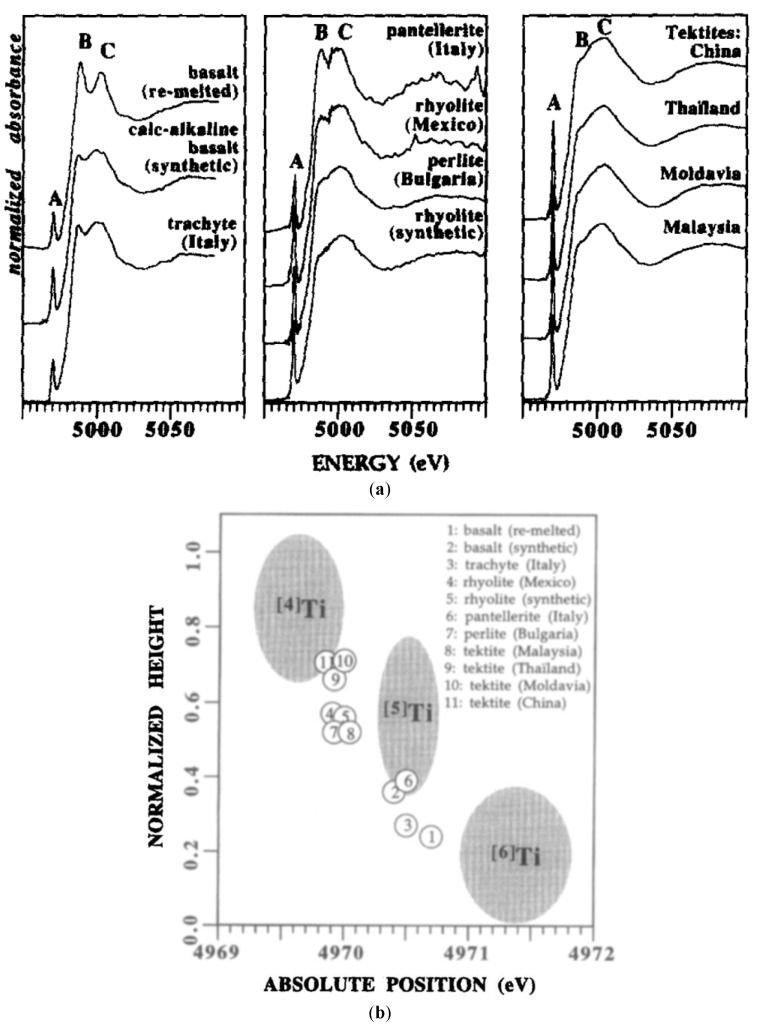
(**a**) Ti K-edge XANES spectra of eleven natural glassy samples and (**b**) pre-edge data for these eleven natural glasses compared to Ti pre-edge data for oxide model compounds (gray areas) in which Ti could be ^[4]^Ti, ^[5]^Ti or ^[6]^Ti [55]. “Reproduced with permission from published by © Elsevier.” (2017).

Farges et al. also studied the short-range order structure around Ti atoms found in seven different types of natural volcanic glasses at ambient temperature and pressure [55]. The energy position and height of the Ti K-edge pre-edge peak of these natural glasses were compared to a set of more than thirty oxide model compounds containing ^[4]^Ti, ^[5]^Ti and ^[6]^Ti coordinations for which the XANES spectra were collected in the same experimental conditions [55]. Figure 8 shows the high-resolution XANES spectra of eleven different natural glassy compositions. They observed that in basaltic, trachytic, rhyolitic and pantelleritic glasses, Ti was present dominantly as (^[5]^TiO)O_4_ units with significant amounts of ^[4]^Ti coordination in the most polymerized compositions as rhyolites glassy samples. Moreover, they observed that for the eleven glasses, the XANES spectra are similar to those of synthetic titanosilicate glasses of simpler compositions such as the 40.3SiO_2_-31.7K_2_O-0.02Na_2_O-26.6TiO_2_ glassy sample measured at 295 K [2,55]. It has also been argued that the increase in the pressure could induce an increase in the number of coordinations of the titanium atom [52]. However, according to the results of Farges et al., the XANES spectrum in the tektite natural glassy sample did not identify any event of pressure increase in this glass. Another important fact observed in this study was that the presence of volatiles such as F, Cl and H_2_O in natural glasses caused few modifications in the coordination of Ti [55]. Another important result derived by Farges et al. from this study was that the ^[5]^Ti coordination transforms to ^[6]^Ti during crystallization of Ti-rich minerals such as ilmenite, rutile and pyrochlore. 

While the chemical coordination was the primary focus of the structural study of titanium ions in different vitreous systems in the study of iron metal ions, the main objective was to study in detail the oxidation state of iron because this element is the most common in natural silicate glasses and magmas, and could exhibit a variable oxidation state, such as Fe^2+^ and Fe^3+^ [43]. Additionally, it has been well established that the Fe^3+^/ΣFe ratio of geological samples can be used as an indicator of redox conditions (or oxygen fugacity) under which a mineral or molten mass is formed [43]. The oxidation state can then be used to infer details about the origin of magma, and also affects physical and chemical properties such as a melt structure, viscosity, volatile component behavior and the temperature and crystallization composition phases [43,58,59,60,61,62]. Furthermore, the addition of iron in synthetics glasses affects its properties such as color, polymerization, viscosity, density and heat capacities of silicate melts [62,63,64]. Iron could be present at dilute levels (few ppm to less than 1 mol % Fe_2_O_3_) in most commercially produced oxide glasses but may also be present at higher levels (more than 1% mol % Fe_2_O_3_) in many glasses resulting from the vitrification of radioactive and toxic wastes and in some melts and glasses of technological and geological interest [65,66,67,68,69,70,71].

The pre-edge peak present at the Fe K-edge XANES spectra, attributed to the *1s*-*3d* transition, has been used to determine the oxidation state and coordination chemistry of iron in silicate and melts [51,63,72,73,74,75]. Additionally, the white line of the Fe K-edge XANES spectra has also been used to determine the coordination number of iron silicate glasses and melts [63,74,75]. 

The first studies where the XAFS technique was used to determine the coordination chemistry and oxidation state of iron in oxide glasses were developed by Chen et al. [19,76]. In these studies, they used a laboratory EXAFS facility to carry out experiments to investigate the Fe^+3^ ions environment in sodium silicate glasses. Based on their EXAFS results, they proposed a structural model of the iron bonding that consisted of three silicon tetrahedrons oriented in such a way that a site was created for the Fe^+3^ ion of *δ* point symmetry, which was consistent with XPS, EPR and Mossbauer studies in the similar silicate glass system [19,76].

Calas and Petiau published a paper where they compared the pre-edge peak of some glasses and crystals with the aim of estimating the four-fold and six-fold ratio of iron sites in these materials [72]. They compared the pre-edge region of the XANES spectra of a Fe^3+^ glass (Na_2_O-2SiO_2_ glass with 3 wt % Fe_2_O_3_) Fe^2+^ glass (0.4MgO-43.4CaO-O.2FeO-SiO_2_) with the pre-edge Fe K-edge XANES spectra of different crystalline minerals compounds. After analyzing their results, they observed that the intensity of pre-edge peaks presents a higher intensity for 4-fold coordination samples and that glassy samples predominantly present this coordination [72]. They also observed an energy shift for a Fe^2+^ glassy sample of mineral compounds, in which they were able to explain the origin.

In 1990, Ziyu Wu et al. published a paper where, for the first time, the multiple scattering (MS) theoretical simulations were used to determine the short-range order in Fe^2+^ and Fe^3+^ glassy samples [75]. They measured the XANES spectra of two natural basaltic glasses and, as was found in previous studies, they determined that the Fe ions are fourfold coordinated by oxygen atoms with a minor contribution of fivefold coordination. Moreover, they observed that the Fe–O bond length in Fe^3+^ and Fe^2+^ glass samples are respectively similar and slightly larger than that of tetrahedrally coordinated iron in crystalline reference samples [75]. From their MS calculations, they obtained results about theFe^3+^/∑Fe ratio similar to those observed by other analytical methods and concluded that their work could be applied to other amorphous systems.

**Figure 9:**
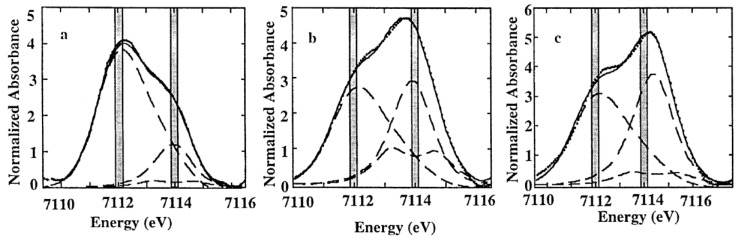
Fe pre-edge region extracted from the K-edge XANES spectra of Volcanic glasses studied by Galoisy et al. [73]. (**a**) Erta Ale basaltic glass; (**b**) oxidized basalt glass and (**c**) Boina pantelleritic glass. The dotted line indicates the experimental spectrum; the dashed lines represent a model for Fe^2+^ in the glass component (augite glass) and ^[4]^Fe^3+^ and ^[6]^Fe^3+^ components. The continuous line represents the fit whereas the shaded zones represent respectively the position of the contribution of Fe^2+^ and Fe^3+^ at 7112 eV and 7114 eV [73]. Reproduced with permission from published by © Elsevier (2017).

The XANES spectra of synthetic and volcanic glasses were studied by Galoisy et al. by using a high-resolution spectroscopy set-up [73]. Using Fe K-edge high-resolution spectra of crystalline reference compounds, they determined the iron oxidation states and site geometry in volcanic glasses (Figure 9) and observed that iron occurs in the ferrous and ferric oxidation states in these samples and it was necessary to consider the contribution of both oxidation states. As Fe coordination geometry affects both the shape and intensity of the pre-edge peak, the pre-edge region could be used to obtain quantitative information on the oxidation state and local environment around Fe^2+^ and Fe^3+^ in volcanic glasses [73].

The authors concluded that the pre-edge XANES spectra of these natural glasses could be reproduced with about 90% confidence using a linear combination between Fe^2+^ and ^4^Fe^3+^ and ^6^Fe^3+^ references samples. This discrepancy was attributed to either the pre-edge extraction or differences in Fe site geometry between the unknowns and model compounds. Additionally, they also concluded that the use of a high-resolution data acquisition was critical to probe the bimodal distribution between both oxidation states and that crucial errors on the quantification of iron oxidation states could be made if site geometry is not taken into account [73].

**Figure 10:**
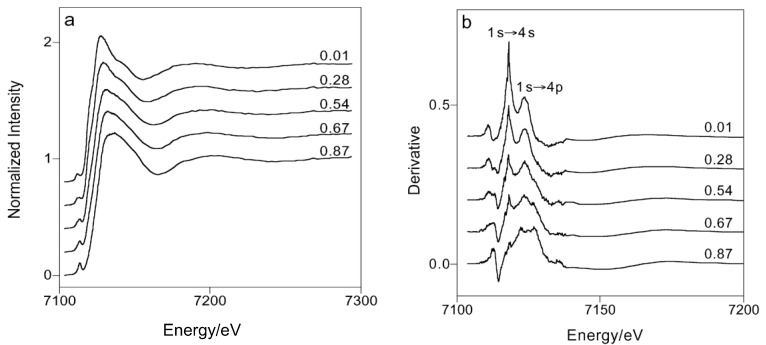
(**a**) Selected Fe K-edge XANES spectra and (**b**) the corresponding first derivative spectra for Fe^3+^/ΣFe ratios [43]. “Reproduced with permission from published by © Mineralogical Society of America.” (2017).

Berry et al. proposed a calibration method that was sensitive to changes in the iron oxidation state but relatively unaffected by variations in iron bonding and the coordination environment [43]. They collected the Fe K-edge XANES spectra for a fully oxidized series and entirely reduced anorthite-diopside eutectic glasses containing 1 wt % 57 Fe_2_O_3_ quenched from melts. Features in the XANES spectra were referenced to the Mössbauer results determine previously to determine their dependence on the Fe^3+^/ΣFe ratio [43]. They tested various methods for extracting Fe^3+^/ΣFe ratios from XANES spectra and found that the energy of the *1s*-*3d* pre-edge transition centroid was found to correlate linearly with the oxidation state of iron atoms. They also observed that there is a correlation with the energy of the K absorption edge and the areas of peaks in the derivative spectra associated with the *1s*-*4s* and crest (*1s*-*4p*) transitions (Figure 10a,b). From Figure 10, they could obtain the energy of the main absorption edge, the area of the derivative peak associated with the *1s* to *4s* transition and the area of the derivative peak associated with the absorption crest (*1s* to *4p*) as a function of the Fe^3+^/ΣFe determined by Mossbauer spectroscopy. They found a deviation from Mossbauer results for end-member spectra (Fe^3+^/ΣFe ~0 and ~1), which they explained by errors arising from the background removal or changes in the Fe^2+^ or Fe^3+^ coordination number with the Fe^3+^/ΣFe ratio [43]. They arrived at the same conclusion as Galoisy et al. [73] who also found that the applicability of any XANES calibration for determining oxidation states is limited by variations in the Fe coordination environment, which affects both the intensity and energy of the pre-edge feature. However, they state that that Fe^3+^/ΣFe values may be obtained from XANES spectra, with accuracy comparable to Mossbauer spectroscopy, by reference to empirical calibration curves derived from compositionally similar standards [43].

**Figure 11:**
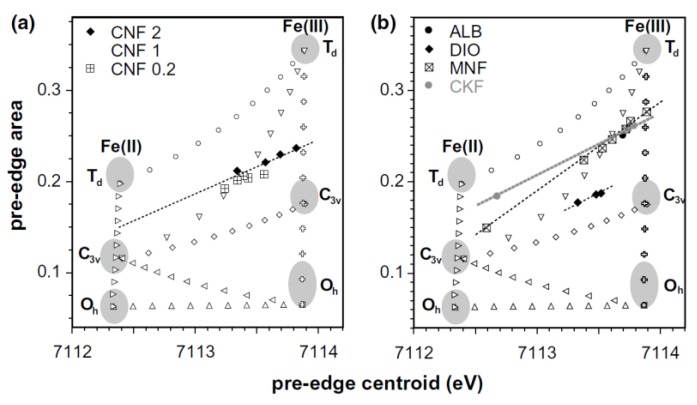
Pre-edge centroid versus pre-edge integrated area for the 27 glassy systems studied by Farges et al. [63]. (**a**) CNF glass samples; (**b**) ALB, DIO, MNF and CKF glasses samples. Sample descriptions in wt %: CNF2 =2.0Fe_2_O_3_-70.9SiO_2_-13.6CaO-13.5Na_2_O; CNF1 = 1.0Fe_2_O_3_-71.5SiO_2_-13.8CaO-13.7Na_2_O; CNF0.2 = 0.2Fe_2_O_3_-72.1SiO_2_-13.9CaO-13.8Na_2_O; ALB = 0.5Fe_2_O_3_-68.4SiO_2_-19.4Al_2_O_3_-11.8Na_2_O; DIO = 1.1Fe_2_O_3_-54.9SiO_2_-25.6CaO-18.4MgO; MNF = 1.0Fe_2_O_3_-74.3SiO_2_-10.3MgO-14.3Na_2_O; CKF = 0.9Fe_2_O_3_-66.7SiO_2_-12.9CaO-19.5K_2_O [63]. Reproduced with permission from published by © Elsevier (2017).

More systematic studies about the influence of oxygen fugacity on the local coordination of iron for a constant glass composition were established by Farges and co-authors [63,77]. They made a high-resolution Fe K-edge XANES study of a series of iron-bearing silicate glasses of simple compositions, which were synthesized at various oxygen fugacities [63]. Moreover, they complemented their studies by a molecular dynamics simulation that was correlated to the obtained structural information. Additionally, they presented a bond valence model that was used to predict the average coordination number of iron as a function of the glass composition. To interpret the structural results from glassy samples, they also measured a series of Fe^2+^ and Fe^3+^ model compounds, which presented different coordination geometries [63]. They analyzed the best function to model the pre-edge peak and they concluded that it was achieved by fixing the width and the amount of Gaussian in a pseudo-Voigt function, which allowed only the position and height to be varied. To model the pre-edge peak, they used a principal component analysis (PCA) and showed that a two-component fits the experimental spectra well and that this result is in agreement with the presence of isosbestic points, which indicated a binary system formed by ferrous and ferric iron with only the oxygen fugacity as the variable parameter. Using this methodology, they could obtain information about the pre-edge centroid energy versus pre-edge area for the studied glasses. They compared it to the information obtained for the Fe^2+^ and Fe^3+^ model compounds, in which Fe ions are located in regular and more distorted polyhedra symmetries. Figure 11 shows the obtained results where the authors also included pre-edge information obtained for some binary mixtures of the end-member compounds used in this study and obtained from a previous study [78]. As can be observed in Figure 11, for all studied glasses, a linear trend in the iron symmetry information as a function of the average iron redox state. For some glassy compositions, they undertook molecular dynamics (MD) calculations in to confirm the behavior observed from XANES analysis and observed that the predicted average Fe coordination number founded by MD calculations was slightly lower than the average experimental value [64]. Additionally, based on a bond valence analysis of these molecular dynamics calculations, a simple model was proposed, which they believed to help predict the speciation of iron in oxide and silicate glasses and melts [63]. 

To provide a more detailed description of the average coordination environment of Fe^2+^ in silica-rich glasses, Jackson et al. adopted a multi- spectroscopy study approach using a ^57^Fe Mössbauer, Fe K-edge X-ray near edge structure (XANES) and extended the X-ray absorption fine structure (EXAFS), UV-Vis-NIR, and magnetic circular dichroism (MCD) spectroscopy [74]. Using these complementary spectroscopy techniques, they characterized the local symmetry and oxidation state of a broad set of glassy samples with different non-bridging oxygen: tetrahedral cation ratios. Several Fe^2+^ and Fe^3+^ model compounds with Fe^2+^ or Fe^3+^ in tetrahedral, trigonal, bipyramidal or octahedral coordination geometries were also studied by XAFS spectroscopy [69]. As in previous studies, they analyzed the pre-edge position to estimate the average oxidation state of Fe as a function of its local environment. They observed that for the ferrosilicate glasses the pre-edge heights are consistent with Fe^2+^ occupying a range of possible sites, which were 4 to 5-fold coordinated on average [69]. The EXAFS spectra of the studied glasses were also analyzed, and a good agreement with XANES results was found with no evidence of a mixture of 4 and 6-fold coordinated Fe^2+^. The authors concluded that all spectroscopic data obtained from XANES, EXAFS, Mössbauer UV-Vis-NIR and magnetic circular dichroism (MCD) for the samples examined were consistent with Fe^2+^ predominantly occupying sites ranging from 5-coordinated, perhaps trigonal bipyramidal, to tetrahedral in their ferrosilicate glasses [74].

Cottrell et al. used high-precision micro X-ray absorption near-edge structure (μ-XANES) measurements to determine the Fe^3+^/ΣFe of natural magmatic liquids [79]. They showed that by using μ-XANES measurements, they could make a direct, in situ microanalysis of Fe^3+^/ΣFe in homogeneous and crystal-free natural magmatic liquids at small scales needed to characterize liquids in natural systems [79]. μ-XANES affords many advantages to bulk techniques as wet chemistry and Mössbauer spectroscopy because of its small spatial resolution (<10 × 10 μm), allowing the oxidation state to be determined on the same spatial scale as other microbeam analytical techniques. They also used the centroid position to quantify the Fe^3+^/ΣFe ratio in minerals and glasses. By analyzing around 16 basaltic reference glasses, they developed a methodology of analysis regarding the factors that could control measurement reproducibility. To do this, they analyzed these basaltic glasses at least three times per synchrotron session and throughout three different sessions to rigorously evaluate this method [79]. Based on these experiments, they addressed various issues related to the utility of μ-XANES in quantifying Fe^3+^/ΣFe from natural basaltic glasses, such as factors controlling session-to-session reproducibility (precision) and the effect of composition on the relationship between the centroid position and Fe^3+^/ΣFe. An important that they showed is that use of a drift monitor permits direct comparison of centroids and Fe^3+^/ΣFe between different beam sessions, and potentially even between synchrotron facilities, which can be considered a fundamental advantage sometimes is impossible to acquire data under the same conditions [79].

Faiz et al. showed the possibility of determining the symmetry and oxidation state of iron in (Fe_2_O_3_)_x_(Na_2_O)_0.30_(SiO_2_)_0.70−x_ (x < 0.2) met-quenched glasses by measuring the O-K and Fe L_2,3_ XANES spectra [80]. These authors argued that the analysis of 2*p* absorption (L_2,3_-edges) spectrum has several advantages over the K-edge spectrum of TM compounds. The L-edge is dominated by dipole transitions from the core 2*p* level to empty 3*d* states, and because of the substantial Coulomb interaction between the two levels, it presents theoretically interpretable multiple structures [80]. Their results showed that Fe atoms in iron-sodium silicate glasses are in octahedral coordination and there is no substantial change in the coordination of Fe as Fe_2_O_3_ substitutes for SiO_2_. However, the hybridization between the O 2*p* and Fe 3*d* states increases as the amount of iron increases. They concluded from the analysis of Fe L_3_-edge spectra that about 10% Fe^2+^ and 90% Fe^3+^ are present in these glasses and that the ligand-field splitting of Fe 3*d* orbital was about 1.6 eV [80].

**Figure 12:**
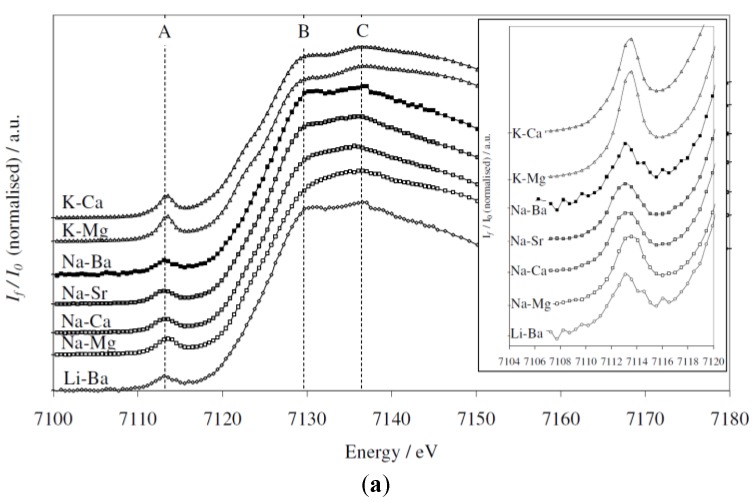

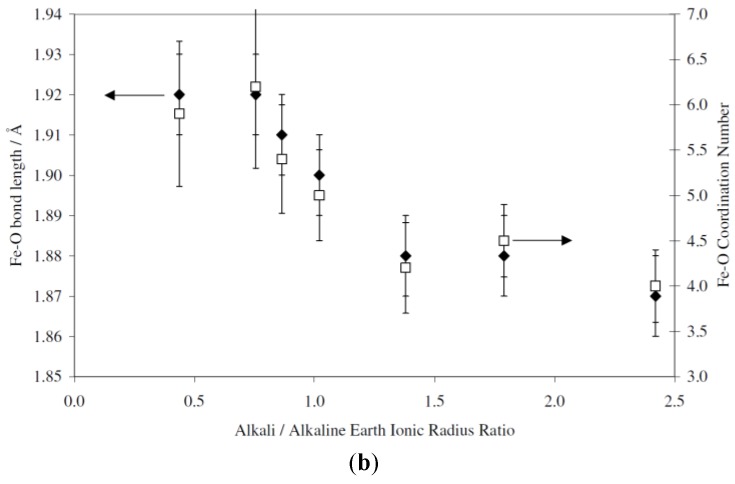
(**a**) Fe K-edge XANES of sample glasses showing changing edge characteristics as a function of the glass composition. Peak A is a pre-edge peak (insert), peaks B and C are edge crest peaks; (**b**) The EXAFS-determined Fe–O bond length and the coordination number as a function of the alkali/alkaline earth ionic radius ratio. Diamond shapes denote the Fe–O bond lengths, and squares denote the Fe–O coordination numbers [67]. “Reproduced with permission from published by © Elsevier.” (2017).

A selective behavior of Fe^3+^ coordination in alkali and alkaline earth cation type in alkali—alkaline earth—silica glasses containing dilute quantities of iron was studied by Bingham et al. [67]. To ensure that all Fe was present as Fe^3+^ ions in their glassy samples, they decreased the melting temperatures combined with doping with an excess of CeO_2_ (0.6 mol %) as an oxidizing agent. This methodology made it possible to quantify the local structural environment of diluted Fe^3+^ in ternary alkali-alkaline earth-silica glasses as a function of the type of alkali and alkaline earth cation [67]. Their XANES results presented in Figure 12a,b show that considerable differences arise in the Fe^3+^ near-edge structure because of the variation in alkali and alkaline earth cation type. They also observed the most substantial effects that occurred within the edge crest (1*s*-4*p*) at ~7129 eV (marked B in Figure 12a) and at ~7136 eV (marked C in Figure 12a) that occurred as functions of the glass composition, which followed different trends as functions of alkali and alkaline earth cation type. Their XANES results confirmed that all Fe was present as Fe^3+^ and that Ce was present as Ce^3+^ and Ce^4+^ [67]. Figure 12b shows the close relationship between the EXAFS-derived Fe–O bond length and average Fe–O coordination number. The bond length increases from ca. 1.87 Å to ca. 1.92 Å and CN decreases from ~6 to ~4 with increasing alkali/alkaline earth ionic radius ratio [67]. Results are consistent with an existing model for the *selective* behavior of Fe^3+^, indicating competition between dissimilar modifier cations or their preferential selection as next-nearest-neighbor cations for Fe^3+^ stabilization [67].

Dyar et al. demonstrated that a multivariate analysis method based on a partial least squares regression used to analyze a full XAS spectra resulted in dramatic improvements in the accuracy of predicting Fe^3+^/ΣFe in garnet samples [81]. Later, they tested the applicability of this result on silicate glasses that are of great interest to geoscientists to overcome the limitations of the previous studies to determine the Fe^3+^/ΣFe ratio [81]. These authors commented that there was considerable diversity in the standards and methods used by different research groups for predicting Fe^3+^/ΣFe in glasses that resulted in a general lack of consistency and accuracy in different studies. For this, they measured and analyzed 372 X-ray absorption spectra from 60 different bulk glass compositions and compared the information obtained from the pre-edge region of these glasses to that in the broader energy range covering the Fe K-edge from 7100 to 7200 eV [81]. They considered their model a robust model, which could determine the Fe^3+^/ΣFe ratio in a wide range of silicate glass compositions. Additionally, they provided software that allowed the calibration to be used on data from any synchrotron that outputs data in some specific formats [81]. They concluded that their work provided a broadly applicable and widely accessible method that is easily implemented using standard XAS file formats and does not require time-consuming fitting of pre-edge features [81].

Very recently, for the first time, Fiege et al. showed results of Fe XANES analyses performed at different synchrotron radiation sources on a broad set of reference glasses and compared their results to the literature [82]. They showed that when they compared XANES spectra collected at different synchrotron beamlines, they observed a negligible effect on the correlation between the centroid energy of the Fe pre-edge peak and the Fe^3+^/ΣFe ratio of the glass samples [82]. They also provided equations for different glass compositions that could be used to calculate the iron valence ratio (Fe^3+^/ΣFe) in glasses by using XANES spectra collected at different synchrotron beamlines. Moreover, they showed that extended exposure to synchrotron radiation does not lead to a detectable change of the Fe oxidation state in the studied silicate glasses, even at the high photon flux density beamlines [82]. Their study confirmed previous studies where they recognized that the effect of bulk composition on the Fe coordination in glasses is governed by changes in the integrated intensity of the Fe pre-edge peak, while the centroid energy remains almost unaffected by variations in composition/Fe coordination [82].

### 4.2. XAFS Studies Concerning the Role of Nucleating Agents in Oxide Glass Materials

The formation of glass-ceramics exhibiting valuable mechanical, electrical and optical properties is mainly dependent on crystal nucleation, and growth and, in some cases, to obtain a glass-ceramic, adding small amounts of nucleating agents is required (heterogeneous nucleation) [83,84,85]. To better understand the mechanism of nucleation and the role of nucleating agents in the nucleation process, it is necessary to follow the process from the formation of the first nuclei, which play a role as a precursor for crystallization, until the complete material crystallization. The precise role of the nucleating agents in the glass-ceramic formation can only be understood by investigating the very first stages of the nucleation process with a strictly local structural probe having atomic selectivity [86]. The XAFS technique has shown to be an appropriate technique to characterize the short-range order structure modification of specific transition metals present in glassy samples from the earliest stage of the crystal nucleation process [44,46,86,87,88,89,90,91,92,93,94,95,96,97,98,99,100]. 

The first ex-situ XAFS studies concerning the role of nucleating agents in the nucleation and crystallization process in silica-based oxide glasses were developed by Dumas et al. [46,101], Petiau and Calas [87] and Ramos et al. [89]. 

Dumas et al. studied the nucleation and crystallization process in SiO_2_-AI_2_O_3_-MgO-ZnO glasses and observed, from the analysis of Zr K-edge EXAFS data, the formation of the ZrO_2_ tetragonal phase, magnesium-petalite and *β*-quartz phases when the glass was heat-treated and the role of Zr as a nucleating agent could not be well established in this case [101]. Ramos et al. analyzed the role of titanium and zirconium in the SiO_2_-Al_2_O_3_-Li_2_O glassy system and observed that titanium coordination changes from 4-fold in a glass to 6-fold coordination in the glass-ceramic whereas the zirconium environment remains highly disordered even in the glass-ceramic sample [89]. 

Using XAFS and Transmission Electron Microscopy (TEM) techniques, the nucleation process in a magnesium aluminosilicate glass (cordierite glass) containing ZnO and TiO_2_ as nucleating agents was studied by Dumas and Petiau [46]. By the analysis of XANES and EXAFS spectra, they observed in the earliest stages of nucleation that important modifications occurred around titanium atoms, which change from four-fold coordination in the glass sample to six-fold coordination in the crystalline sample. On the other hand, zinc atoms remain in four-fold coordination during the crystallization process with a higher degree of a short-range disorder that decreased as the sample became crystallized. By the analysis of EXAFS data, they observed that the second neighbors around zinc are predominantly zinc and titanium atoms meaning that these atoms are the primary elements present in the phases that were first crystallized [46].

Meneghini et al. showed the complementarily of ex-situ X-ray scattering (XRS), X-ray absorption spectroscopy (XAS) and differential anomalous X-ray scattering (DAS) experiments to follow the crystallization process of a CaO-SiO_2_-ZrO_2_ glassy system when submitted to a heat treatment [90]. Due to the complementarily of these techniques, the evolution of the long-, medium- and short-range structures as a function of the heat treatment could be followed, in particular concerning the Zr environment. They observed that the quartz phase starts to crystallize first, followed by crystallization of the wollastonite phase and that the Zr-Si and Zr-Zr coordination numbers increase, indicating a progressive ordering of the Zr environment with temperature. Due to a large number of Zr-Si next neighbors observed in the fitting procedures, they assumed that a zirconium silicate phase was formed. Finally, they concluded that their data provided evidence that the system presents, at least, independent crystallization paths for wollastonite, for quartz and for a zirconium silicate [90].

**Figure 13:**
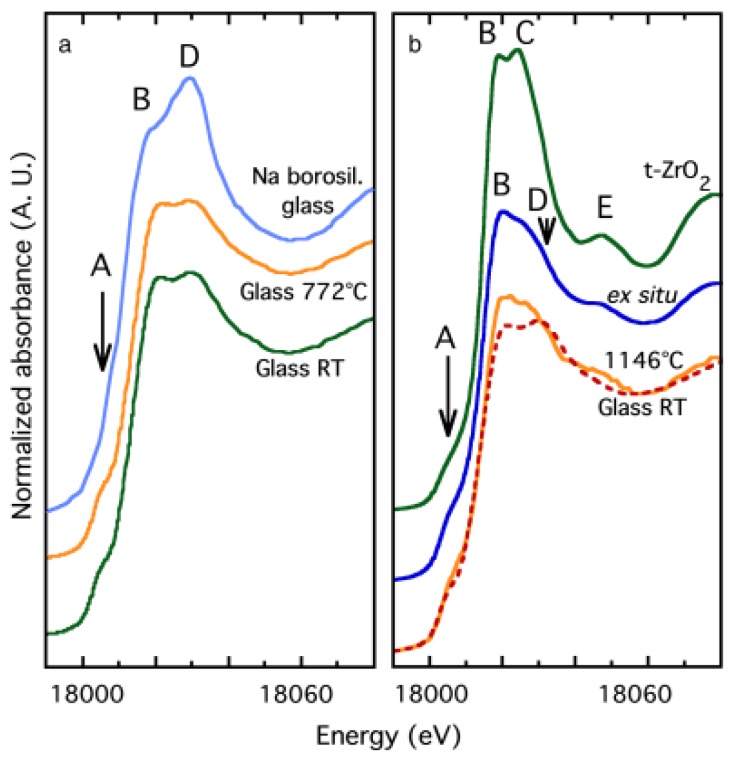
Zr K-edge XANES spectra of: (**a**) glassy sample measured at room temperature (Glass RT), glassy sample measured at 772 °C (Glass 772 °C), alkali borosilicate glass (Na borosil. glass); (**b**) glassy sample measured at room temperature (Glass RT), in situ nucleated glass at 1146 °C (1146 °C), ex situ nucleated glass (ex-situ) and Y-stabilized tetragonal ZrO_2_ phase (t-ZrO_2_) [93]. “Reproduced with permission from published © John Wiley and Sons.” (2017).

An in situ studies of nucleation of the zirconia phase in an MgO-Al_2_O_3_-SiO_2_ glass was presented by Dargaud et al. [93]. Figure 13a shows the XANES spectra of glassy samples collected at room temperature and below the transition temperature compared with a XANES spectra of an alkali borosilicate glass whereas Figure 13b compared the XANES spectra of the room temperature glassy sample and tetragonal t-ZrO_2_ crystalline phase with an in situ and ex-situ nucleated glassy sample. By analyzing the Zr K-edge XANES spectra, mainly based on modifications observed at the edge region, they observed that in the glassy sample, Zr atoms are coordinated by a higher number of oxygen atoms compared with other glass systems, which explained the structural stability of Zr during thermal treatment. In the nucleated glass, the Zr environment precludes the presence of Zr in the residual glassy matrix and demonstrates that the nano-ZrO_2_ particles still exist in the final glass-ceramics [93]. These authors found a similar result in a similar study and showed that there is only a small evolution of the short-range order around Zr atoms during the first stages of nucleation that demonstrates the major role played by the structural properties of Zr as a nucleating agent [91].

Cormier et al. used the X-ray absorption spectroscopy at both Ti K- and L_2,3_-edges to investigate the role of TiO_2_ as a nucleating agent in the 2MgO-2Al_2_O_3_-5SiO_2_ + xTiO_2_ with x = 0, 2, 4, 6, 8, 10 mol % glassy system [92]. They added different amounts of TiO_2_ and submitted the glassy samples to different thermal treatment that led to the surface or bulk crystallization. Using the correlation between the coordination environment around Ti and the pre-edge parameters (position and height) established by Farges et al. [2,45], they determined the coordination environment of Ti atoms in glassy and glass-ceramic samples. During the nucleation, the L_2,3_-edges spectra underwent major changes that were compatible with Ti coordination changes from mainly a ^[5]^Ti to mainly ^[6]^Ti environment. For the first time, they also observed modification in the Ti environment between the nucleation front and the crystallized part and showed the presence of a specific interface between the crystalized region and the vitreous bulk in a ceramic glass sample. This result indicated that the structure was modified as the crystallization proceeds because they found a different Ti environment between the initial nucleation region and in a region of an advanced stage of crystallization, implying that modifications of the glassy matrix, both structural and compositional changes, alter the nucleation pathways. This emphasizes that not only the crystallized part but also the glassy part, must be investigated to fully understand and control the formation of glass ceramics [92].

Using a combination of Zr L_2,3_-edges XANES spectra with STEM and EDX analyses, Patzig et al. also studied the role of Zr^4+^as a nucleating agent in MgO-Al_2_O_3_-SiO_2_ glass ceramics [94]. They observed changes in the coordination number of zirconium atoms from a ^[6]^Zr^4+^ in the glassy sample to ^[8]^Zr^4+^ after the thermal treatment; the eight-fold Zr coordination was associated with the formation of nano-crystalline tetragonal ZrO_2_ phase [94]. 

The origin of the color and crystallization process of ZrO_2_/TiO_2_ doped Li_2_O-Al_2_O_3_-SiO_2_ (LAS) glass system as a function of the TiO_2_ amount was studied by Chavoutier et al. using XAFS and other micro structural and structural techniques [97]. By analyzing the XANES data collected at the Ti K-edge, they observed that Ti^4+^ ions have the same average environment for the studied range of compositions and that titanium presents a mixture of fourfold and fivefold coordination in these glasses. The analysis of the Zr K-edge XANES spectra showed that Zr short-range order is close to that of the ZrTiO_4_ phase with a distorted ZrO_6_ environment, but also with the possible presence of a more regular 6-fold coordination site for Zr^4+^ ions [97]. After a heat-treatment, Ti K-edge XANES spectra indicated that the average Ti coordination in the glass-ceramic increases and is attributed to a greater fraction of Ti^4+^ cations in six-fold coordination. Finally, based on the results analysis of the different techniques that they used, they concluded that the color of the glass is due to Ti^4+^-O_2_ charge transfer and not to the presence of Ti^3+^ ions [97]. 

In 2014, Cormier et al. presented an excellent paper where they showed selected results about the structural characterization of the environment around Zr, Ti and Ni ions in glasses and glass-ceramics using the XAS technique, trying to understand the role of each of these ions in the nucleation/crystallization process [96]. Due to the active role of these ions on the nucleation/crystallization process, to better understand this process, structural modifications around these ions in the earliest stages of nuclei formation should be followed [96]. Based on some examples, they showed that there is no clear relationship between the local site geometry and the propensity to favor bulk nucleation and that in these cases, the knowledge of medium-range order organization is essential to understand the nucleation process with evidence of heterogeneities or structural fluctuations that can be directly related to the first crystallizing phases [96]. They also showed the importance of in situ measurements using a lower heating rate and the sudden modification observed in the XANES and EXAFS data showed that the very initial stages of the nucleation could be probed mainly when XAS methods were coupled with other techniques such as XRD and DSC [96]. Finally, in this paper, they also showed that to gain more information on the nucleation/crystallization mechanisms, it is crucial to analyze the formation of nanometric crystals with the available nano- or micro-XAS beamlines, as well as timely resolved in situ high temperature XAS measurements on synchrotrons that opened up new opportunities to obtain spatially and timely resolved structural information [96].

**Figure 14:**
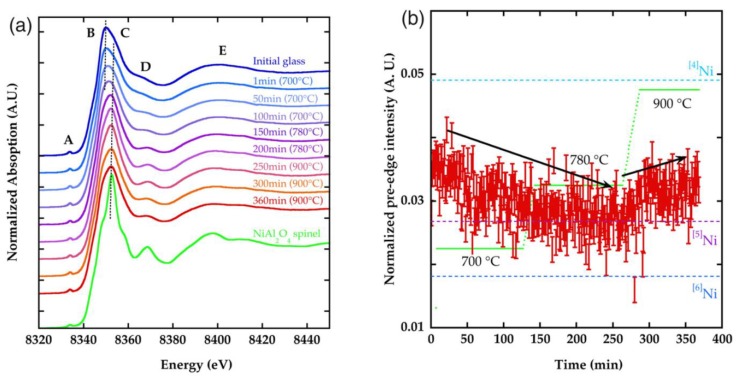
(**a**) Ni K-edge XANES spectra as a function of temperature and time for the Li_2_O-Al_2_O_3_-SiO_2_-1.7NiO glass compared to the room temperature spectrum of NiAl_2_O_4_ crystalline phase; (**b**) Evolution of the pre-edge intensity of the XANES spectra shown in Figure 14a. Green line: heating ramps and temperature isotherms; horizontal lines indicate the pre-edge intensity for Ni in different coordination sites in crystalline reference compounds [102,103]. “Reproduced with permission from published by © Elsevier.” (2017).

Dugue et al. studied in detail the structural evolution of Ni ions in lithium, magnesium, and zinc aluminosilicate glasses and glass-ceramics containing TiO_2_ and ZrO_2_ as nucleate agents [102,103]. In situ high-temperature XAS measurements, which allowed the determination of the Ni^2+^ environment and its evolution as a function of time and temperature, showed that crystallization sequences are strongly dependent on glass compositions [102,103]. Figure 14 shows, as an example, the Ni K-edge XANES spectra and the evolution of the pre-edge intensity of a Li_2_O-Al_2_O_3_-SiO_2_ (LAS) glass sample containing nickel measured in different isotherms and times. Significant modifications on the XANES spectra were observed when the sample was measured at 700 °C after 50 min with the appearance of a contribution similar to that observed in the NiAl_2_O_4_ spinel crystalline phase XANES spectrum, that corresponded to a ^[6]^Ni^2+^ coordination. As the temperature increased, the XANES spectra become closer to that of the spinel phase although broader features are observed due also to the presence of Ni^2+^ in the residual glassy phase. They state that the formation of a spinel phase implies a ^[5]^Ni^2+^ to ^[6]^Ni^2+^ transformation that could be confirmed by a reduction in the pre-edge height, however, due to phono-electron interactions that increase the height as the temperature is raised, the quantitative analysis of Ni^2+^ coordination is subject to uncertainties [102,103].

Recently, Cormier et al. made a comparison of the role of the Zr element on the nucleation/crystallization process about a Zr-free glassy sample by using Zr K-edge and Zr L-edge XANES spectra [99]. Their results showed that Zr coordination could not be a relevant parameter to establish the nucleation role of Zr as they observed similar Zr local coordination in glasses where nucleation occurred and did not occur. They concluded from their EXAFS results that the medium range ordering of the Zr sites appeared to be a critical parameter in defining the nucleating properties of Zr-bearing glasses [99].

**Figure 15:**
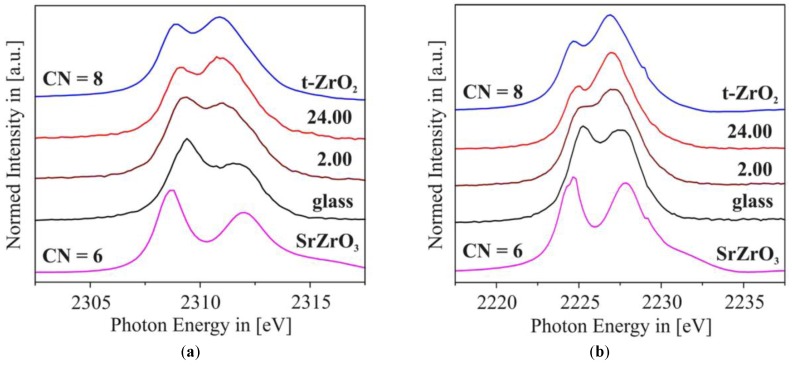
(**a**) Zr L_2_-edge XANES spectra and (**b**) Zr-L_3_ edge XANES spectra of LAS glass-ceramic sample compared to the spectra of the glassy sample and ^[6]^Zr^2+^ and ^[8]^Zr^2+^ crystalline reference compounds [104]. “Reproduced with permission from published © Nature Publishing Group.” (2017).

Very recently, Kleebusch et al. presented a detailed study about the crystallization behavior of Zr as a nucleating agent in Li_2_O-Al_2_O_3_-SiO_2_ (LAS) using a combination of different techniques such as X-ray absorption fine structure spectroscopy (XAFS), X-ray diffraction (XRD) and Scanning Transmission Electron microscopy (STEM) [104]. These authors used the fact that the intensity ratios of the doublet peaks of both the Zr L_2_-edge and Zr L_3_-edge are dependent on the coordination number to determine the local coordination around Zr atoms [105]. Figure 15 shows a comparison of Zr L-edges XANES spectra of LAS glass containing zirconia with the XANES spectra of the sample at the final stage of heat-treatment, as well as the XANES spectra of crystalline reference compounds where Zr atoms are sixfold ^[6]^Zr^4+^ in SrZrO_3_) and eightfold coordinated ^[8]^Zr^4+^ in t-ZrO_2_ [104]. From this figure, it is clear that in the glassy sample, Zr ions are mainly six-fold coordinated by oxygen atoms whereas, at the final stage of heat-treatment, the XANES spectra of the glass-ceramic sample are similar to the XANES spectra of the t-ZrO_2_ where Zr ions are eightfold coordinated by oxygen atoms. 

### 4.3. XAFS Studies Concerning the Relationship between the Short-Range Order and the Nucleation Tendency in Silicate Glasses 

Different studies have tried to establish a relationship between the similarity of the parent glass and its isochemical crystal structure at the molecular level (local order around the network modifier_cations) and their tendency to nucleate in the volume or at the glass sample surface [106,107]. The central assumption is that when small rearrangements occur at the interface of the two phases to start the nucleation process, nucleation inside the sample will occur more efficiently, i.e., if the short-range order of glass and crystal is similar. If the structural differences are substantial, the nucleation process will only take place on the sample surface/air, or in the volume with the aid of nucleating agents [106,107].

**Figure 16:**
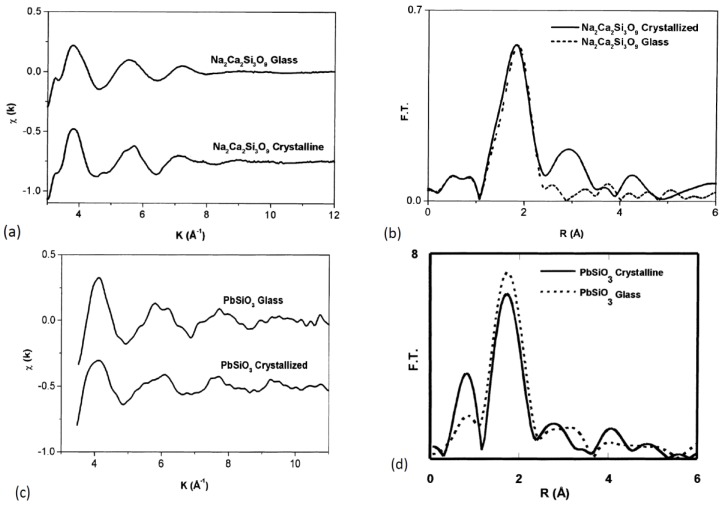
(**a**) Ca K-edge EXAFS spectra and (**b**) corresponding Fourier transform of glassy and crystallized Na_2_Ca_2_Si_3_O_9_ sample. (**c**) Pb L_3_-edge EXAFS spectra and (**d**) corresponding Fourier transform of the glassy and crystallized PbSiO_3_ sample [108]. “Reproduced with permission from published by © Elsevier.” (2017).

To the best of our knowledge, only one study developed by the present author, using the XAFS technique, proposed a clear relationship between the short-range order of selected ions in glassy silicate systems and the nucleation tendency [108]. The XAFS technique was used to study the short-range order of the network modifier ions in three glassy systems: CaSiO_3_, Na_2_Ca_2_Si_3_O_9_, which presented volume nucleation, and the PbSiO_3_ system, for which the nucleation occurs only on the glass sample surface. As an example, Figure 16 shows the EXAFS spectrum and respective Fourier transform of the Na_2_Ca_2_Si_3_O_9_ glass—which crystallizes homogeneously in the volume—and the EXAFS spectrum and respective Fourier transform of the PbSiO_3_ glassy sample, which crystallizes homogeneously in the volume [108]. By analyzing the EXAFS results, it could be inferred that in glasses that have a high tendency to volumetric nucleation (CaSiO_3_ and Na_2_Ca_2_Si_3_O_9_), the short-range order structure of the network modifying cations is similar to the short-range order of their isochemical crystalline phases. On the other hand, the short-range order of the lead atoms in the glass that has a low tendency to volumetric nucleation (PbSiO_3_) is different from the short-range order in the crystalline phase of the same composition.

Although we could not find other studies dealing directly with the application of the XAFS technique to propose a correlation between the nucleation mechanism and the short-range order structure in glassy materials, some authors have published a review about this statement and analyzed a wide range of structural results obtained at different structural scales [109,110]. According to Deubener, based on an extensive analysis of a vast range of glassy systems and structural results, at short and medium range orders, although the short-range structure in some stoichiometric silicate glasses and isochemical crystals are very similar, there is no a relationship with the fact of the ease of those glasses to nucleate in the volume [109]. On the other hand, according to Zanotto et al., a thorough re-analysis of structural data for several stoichiometric oxide glasses and their respective isochemical crystals reveals a clear positive correlation between the homogeneous nucleation ability and the structural similarity at the level of short- and intermediate-range orders of the network modifier cations [110].

### 4.4. XAFS Studies Concerning the Short-Range Order Structure of Transitions Metals in Oxide Glasses as a Function of the Temperature

The structural environment of transition metals in an oxide melt above the liquidus temperature is not necessarily the same as that in a quenched glass, which was frozen at around T_g_, although it is often assumed to be. To study this problem directly for geochemically relevant melts, the XAFS technique was applied to study the local environment of transition metals such as Fe, Ti, Cr, Mo and Ni in oxide glasses [43,59,111,112,113,114,115,116,117,118,119,120,121]. The response of disordered systems such as glass and supercooled liquids and melts to increasing temperatures, particularly above T_g_, is more difficult to predict because of the variable behavior of the various components found in the glass during heating [111]. Among the different transition metals cited above, the temperature effect on the short-range order structure was studied in more detail in oxide glasses containing Ti, Fe and Ni. Iron is considered the most important transition element in natural silicate melts and occurs in two valence states, Fe^2+^ and Fe^3+^. Their relative abundances depend markedly on thermodynamic variables and composition. Most properties are strongly affected by the total iron content and iron speciation, with the consequence that crystallization, viscous flow and other physical processes in natural and industrial settings also depend on the redox state [118,121]. Ni^2+^ is an important trace element in silicate minerals and melts of the deep Earth, and is known to partition strongly from the melt to coexisting minerals such as olivines and pyroxenes [113,122].

One of the first studies about the dependency of the short-range order structure of Fe^2+^ on silicate glasses and melts was developed by Waychunas et al. [111]. By analyzing Fe short-range order in Na_2_FeSi_3_O_8_ and K_2_FeSi_3_O_8_ soda silicate glasses and melts, they concluded that Fe^2+^ is within a four-coordinated network of oxygen in these glass/melt systems. The structural data for Fe_2_^2+^SiO_4_ melts suggest the possibility of a pressure-induced change from four to six-coordination pressure for Fe^2+^ in magmas in the Earth´s upper mantle [111].

The short-range order structure around Ni on a Na_2_Si_2_O_5_ glass containing 2 wt % of NiO glass and melt was studied by Farges et al. [113]. From the measurement of EXAFS spectra at Ni K-edge collected in temperatures between 293 K and 1250 K, they observed that Ni-O distances for the glass at 293 K are consistent with a mixture of ^IV^Ni and ^V^Ni, whereas the melt has dominantly ^IV^Ni [113]. From these data, the authors concluded that the significant reorganization of the melt during quenching helped to explain the enrichment of Ni in early-formed igneous minerals and the possibility of a pressure-induced coordination change around Ni, which may significantly influence the geochemical modeling of the Earth [113].

After establishing a good correlation between the pre-edge peak position and height with titanium coordination in a large set of Ti-bearing glassy systems [45], Farges et al. analyzed the short-range order structure around Ti atoms in five titanium silicate glasses with TiO_2_ concentrations ranging from 2.7 to 30.5 wt % by in situ XAFS measurements at temperatures ranging from 293 to 1650 K [52]. Their objective by measuring the short-range order structure around titanium was to confirm or not the proposal made by Lange and Navrotsky that changes in the Ti coordination number may be a possible structural explanation for the anomalous variation observed in heat capacities in Na and K-bearing titanosilicate glasses in temperatures just above the glass transition temperature, T_g_ [52,57]. 

The first significant result of these in situ measurements was the verification that the analysis of the pre-edge peaks in the studied glasses and melts could be carried out without considering the effects of temperature (anharmonicity) on the oxygen distribution around titanium atoms [52]. On the other hand, they showed that it is necessary to consider an a harmonic model to correctly analyze both positional and thermal disorder effects obtained from the EXAFS spectra [52].

**Figure 17:**
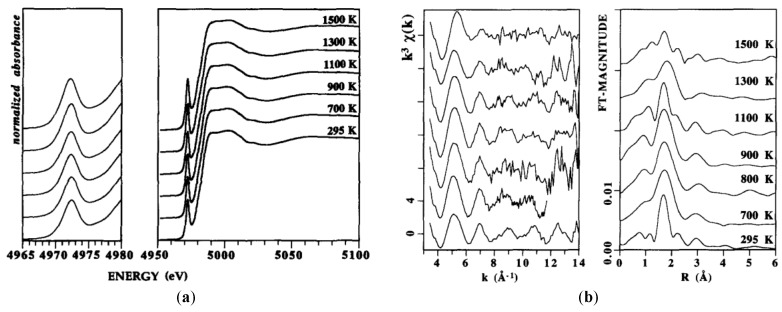
(**a**) Pre-edge (left) and XANES spectra (right) collected at Ti K-edge of NTS2 (46.1SiO_2_-0.07K_2_O-24.0Na_2_O-30.6TiO_2 _wt %) glass and melt; (**b**) Normalized k^3^ weighted EXAFS spectra (left) and Fourier Transform (right) of the same glass [52]. “Reproduced with permission from published by © Elsevier.” (2017).

Figure 17 shows, as an example, the XANES, the EXAFS and respective Fourier transform of NTS2 glass (46.1SiO_2_-0.07K_2_O-24.0Na_2_O-30.6TiO_2_ wt %) measured at temperatures ranging from 295 to 1500 K [52]. The authors observed that the changes due to the increase in temperature above T_g_ (~860 K) are small, which suggest little modification in the disorder during the glass to the supercooled liquid transition. The increased pre-edge peak height from 0.43 to 0.52 up to T_g_ suggested an increase in the ^[4]^Ti coordination in the melt compared to the glass. This result was confirmed by the fitting of the EXAFS spectra, which showed that the short-range order structure regarding Ti-O (Si) bonds does not vary significantly with the temperature [52].

In 1999, Farges et al. proposed a new device to collect in situ high-temperature XAFS spectra at low X-ray energies from the K edge of Na to Cl [120]. XAFS data were collected in the fluorescence mode using a heated loop that contains the sample. A Na_2_Si_2_O_5_ glass was studied at the Si K-edge from 293 to 900 K. From these experiments; the authors showed the possibility to collect data at high temperatures at low energies using the fluorescence mode of different complex glassy oxides containing relatively light elements such as Mg, Na, AI, Si [120].

**Figure 18:**
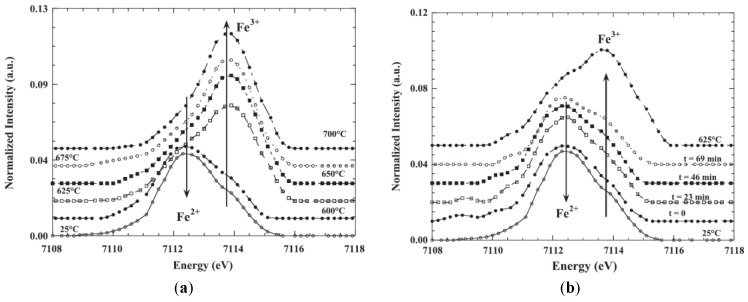
Evolution of the Fe K-edge pre-edge peak with temperature and time for PyrNa17R glass (52.98SiO_2_-11.99MgO-17.00CaO-5.48Na_2_O-12.75FeO): (**a**) collected at different temperatures; (**b**) collected at 600 °C at different times in comparison with the pre-edge data collected at 25 °C and 625 °C [59]. “Reproduced with permission from published by © Elsevier.” (2017).

A controlled atmosphere furnace constructed by Berry et al. was used to determine the oxidation state of chromium in the presence of iron ions at temperatures up to 1773 K [123]. By analyzing the Cr K-edge, for the first time, they showed the presence of Cr^2+^ and Cr^3+^ in a Fe-bearing melt and that Cr^2+^ oxidizes to Cr^3+^ on cooling in the presence of Fe^3+^. They concluded that their research provides the first direct evidence for the presence of Cr^2+^ in terrestrial basaltic magmas and shows the importance of in situ determination of metal oxidation states in melts [123]. Magnien et al. collected Fe K-edge XANES spectra of supercooled melt of Fe-bearing pyroxene samples to study the kinetics of iron oxidation at temperatures below and above the glass transition temperatures [59]. Figure 18 shows the evolution of the Fe K-edge pre-edge peak with temperature and time for PyrNa17R glass (52.98SiO_2_-11.99MgO-17.00CaO-5.48Na_2_O-12.75FeO) heated in air from 25 to 700 °C [59]. They observed a small difference between the spectra recorded at room temperature and 600 °C, below the glass transition. The evolution is almost complete in the 700 °C pre-edge spectrum where the low-energy shoulder has nearly disappeared. Their XANES experiments showed that the kinetics of iron oxidation did not change much with the temperature, even at temperatures below the glass transition (608 °C). They concluded that the rate-limiting factor in this process is not oxygen diffusion, which is coupled to the relaxation of the silicate network, but, diffusion of network modifying cations along with a counter flux of electrons [59,124,125,126].

In 2007, Wilke et al. provided new information about the incorporation of Fe^2+^ and Fe^3+^ into a variety of binary alkali-silicate [117]. These investigations were used to propose a relationship between Fe speciation and bulk composition, focusing mainly on the influence of the number of non-bridging oxygen atoms per tetrahedron [117]. They used a more appropriate normalization procedure for the XANES region based on a broad energy range (about 250 eV in total) before and after the Fe K-edge [117]. These authors observed that in most of the studied samples, the high-temperature XANES spectra differed from those of the quenched samples, mainly at oxidizing conditions, where glasses and melts displayed a more heterogeneous behavior. They concluded that the amount of non-bridging O atoms in the glass/melt system play an essential role, particularly for Fe^3+^, however more complex relationships between Fe and other structural components, especially aluminum, are possible [117].

Magnien et al. made use of Raman and XANES spectroscopies sensitivity to the Fe^3+^/ΣFe ratio to determine the influence of the nature of alkali cations on the redox kinetics near the glass transition range (Raman), as well as at superliquidus temperatures (XANES) of oxidized and reduced samples of three different melts [118]. The same kinetics were observed with both techniques described by characteristic times that depend primarily on temperature and not on the initial redox state. At high temperatures, where both kinds of reactions were investigated, these times were similar for oxidation and reduction processes [118]. From these characteristic times, the authors calculated as a function of temperature and composition a parameter termed effective redox diffusivity and observed that the diffusivities followed two distinct Arrhenius laws, which indicated that the mechanisms of the redox reaction are not the same near the glass transition and at high temperatures. Based on their results, they established that the diffusion of divalent cations is the dominant mechanism at low temperatures but the enhanced kinetics observed for alkali-bearing melts indicated that Li^+^ and Na^+^ also participated in ionic transport [118]. At superliquidus temperatures, in contrast, oxygen diffusion represents the dominant mechanism [118].

Very recently, aerodynamic levitation and laser heating system were used to characterize the local structure and oxidation state of iron ions in molten materials [119]. Their research demonstrated that precise information could be obtained from XAS data of levitated melts, even at lower energy absorption edges if the stability and symmetry of the sample and self-absorption effects are considered. Comparisons between melts and their corresponding glasses reveal that pre-edge peak areas change little in quenching, implying that the Fe–O coordination is only weakly temperature dependent. The authors concluded that the aerodynamic levitation and laser heating methodology provided a powerful tool to characterize the local structure and oxidation state in molten materials over a wide temperature range spanning super-cooled liquids. Since the effect of heterogeneous nucleation is minimized, liquids at extreme temperatures exposed to a wide range of oxygen fugacity can be studied without having the typical problems encountered when using solid containers [119].

### 4.5. XAFS Studies Concerning the Short-Range Order Structure of Glasses at High Pressure, Nuclear Waste Glasses, and Medieval Glasses

Structural and physical properties of silicate melts are very important for the fundamental understanding of magmatic processes [127,128]. At high pressures and temperatures, coordination number (CN) changes of Si in silicate melts could occur and probably result in drastic changes in some physical properties, such as viscosity [128]. The structural mechanisms related to this process are still unclear. Germanate glasses have been considered as structural analogs of silicates, and since they show phase transitions at a lower pressure than silicates, they can be used as model compounds, which are more accessible to high-pressure experiments [128]. One of the first studies about the pressure effect in silicate glasses was developed by Fleet et al. [127]. The effect of pressure in short-range order structure of germanates-based oxide glasses as, GeO_2_, Li_2_O-4GeO_2_, SiO_2_-GeO_2_ and La_2_O_3_-B_2_O_3_-GeO_2_ glass systems have been studied by different authors [128,129,130,131,132,133,134]. Recently, Mouton et al. studied the high-pressure structural changes in diopside glass by analyzing oxygen K-edge XANES data and revealed that diopside shows a clear structural transition around 4 GPa and a probable second transition between 12 and 14 GPa [135]. We also found in the literature pressure-induced structural changes where the XANES spectra of K_2_TiSi_4_O_11_ silicate glass was collected from the samples quenched from different pressures [58]. The XANES spectra collected at the Ti K-edge showed a variation with the pressure that was related to changes in the geometrical environment around the Ti atoms [58]. By comparing it with the XANES spectra of crystalline materials, they observed that the glass sample presented a relatively low average coordination number near to 5 in samples quenched at low pressure and a higher coordination number near to 6 in samples quenched from the highest pressure [58].

The XAFS technique has also been widely used to study the short-range order and electronic structure of different atomic species in nuclear waste glass materials because vitrification of high-level radioactive waste (HLW) in borosilicate glasses is used in various countries [136,137,138,139,140,141,142,143,144,145,146,147,148,149,150,151,152,153]. The radiation effects on the stability and durability of these waste forms have been actively pursued, and different studies have focused on the basic understanding of the radiation-damage process [141]. XAFS is an ideally suited technique to address these effects because it is sensitive to the changes in short-range order that accompany the effects of internal/external radiation damage [141].

Very recently, McKeown et al. studied (by XAFS) the role of vanadium addition on the structure of 44 different systems of molybdenum-based glasses [152]. According to these authors, the presence of a significant amount of molybdenum in the glasses can lead to the formation of an unwanted molybdate salt phase that is considered a primary factor that limits waste loading in the HLW glass product [152]. X-ray absorption spectroscopy (XAS) and Raman spectroscopy were used to characterize Mo environments in HLW borosilicate glasses and to investigate possible structural relationships between Mo and V. The analysis of Mo XAS spectra indicated isolated tetrahedral Mo^6+^O_4_ with Mo-O distances near 1.75 Å whereas V K-edge XANES spectra indicate tetrahedral V^5+^O_4_ as the dominant species. Based on XAS and Raman results, these authors commented on the possible formation of MoO_4_-MoO_4_ and MoO_4_-VO_4_ clustering, that suggested that V additions could stabilize Mo in the matrix concerning the Mo yellow phase formation [152].

The XAFS technique has also been used to study ancient and medieval glasses [154,155,156,157,158,159,160,161,162,163,164]. Different studies focused on the determination of the origin of color in ancient and medieval glasses because this kind of information is important to understand the manufacturing technique of ancient glassware and potteries [154,155,158,159,160,165]. Recently, Farges and Cotte published a chapter in a book where they update as compared to the most recent review papers published on the application of XAFS technique to study cultural heritage [166]. 

Among different studies where XAFS was used to study medieval glasses, we can cite, for example the work by Ferrand et al. [160] that analyzed by XAFS three pieces of historical on-site glass windows dated from the 13th to 16th century and one archeological sample from the 8th century that showed Mn-rich brown spots at their surface or subsurface [160]. Based on XAFS results, they observed that the oxidation state of Mn is different compared to fresh glass confirming that a modification of the Mn environment occurred. No precise identification of the crystalline phase modification was possible from a fingerprint analysis of XANES spectra whereas EXAFS data analysis led to identifying a Mn environment in this crystalline phase modification [160]. 

The knowledge of the chemical composition, chromophores and surface structure is important for both archaeological glasses and stained glass windows because it helps to complete their historical knowledge, such as provenance, dating and authentication studies; to optimize restoration and conservation procedures and improve preservation strategies, such as storage or exhibition conditions [164]. Abuin et al. [164] carried out a study on the historical original glass chromophores as indicators of their decay with the aim of establishing a relationship between the oxidation state of Fe, Cu and Mn ions, the molecular environment and their degradation state by studying both the XANES and EXAFS regions [164]. These authors found out that it was possible to establish a relationship between the oxidation state of Fe and Cu with the decay presented by the glass. Their results indicated that the Mn-oxidizing state was not directly involved in the glass decay in the studied samples [164]. 

To conclude this review, it is important to draw attention to a recently proposed definition of glass [167] “*Glass is nonequilibrium*, *non-crystalline condensed state of matter that exhibits a glass transition. The structure of glasses is similar to that of their parent supercooled liquids (SCL)*, *and they spontaneously relax toward the SCL state. Their ultimate fate*, *in the limit of infinite time, is to crystallize.*” This definition describes the vitreous or glassy state of matter with the following concepts: non-equilibrium state, non-crystalline structure, relaxation (T_g_) and crystallization. In this review, we have shown that XAFS is a handy tool to characterize glass structure and crystallization phenomena; critical features of the glassy state. 

## 5. Summary

Significant progress in the quality of the obtained XAFS data and, consequently, on the interpretation of results, has occurred over the past 45 years due to improvements in the experimental setup used to collect data regarding energy resolution, time-resolved measurements, using micro and nano-probes, improved theoretical models and software. Advances in synchrotron beamlines have enabled researchers to analyze samples in situ, which was not possible before. It is also important to mention the more recent possibility of modeling the XANES and EXAFS spectra of disordered materials using *ab initio* multiple-scattering codes, such as f, allowing for a better interpretation of XANES spectra at short and medium range order. It is also relevant that, in many cases, successful interpretation of XAFS data was achieved using complementary advanced structural techniques, such as Raman, X-ray and neutron scattering, Mossbauer spectroscopy, UV-Vis-NIR, and magnetic circular dichroism (MCD) spectroscopies. 

We have selected and reviewed some studies including many published to date on the structural role of Ti, Fe and Ni transition metal ions in different oxide glass properties. Our analysis shows that, in recent years, more attention has been paid to studies on synthetic or natural oxide glass containing iron ions. This is due to the higher interest by researchers of different fields to have a better understanding of the physical and chemical processes that occur in the transformation of a liquid to the vitreous state and then to the crystalline state, for example, of magmas. A recently published study on the use of a sample levitation system (avoiding contact with the container during melting) is a breakthrough in this area of research.

Regarding nucleation and crystallization studies in glasses, recent studies have shown that the role of nucleating agents should be related to a structural order of glasses at a medium distance. Several examples presented here show that it is possible to obtain information of medium-range order through XANES spectra analysis. It is important to note that X-ray absorption spectrum measurements at low energies allow only the XANES spectrum to be obtained in most cases, and in situ experiments are difficult to perform due to experimental restrictions. Using other techniques to obtain quantitative information about medium-range order, such as the atomic pair distribution function (PDF) approach, to study the local structure of liquids, glasses and disordered crystals, may provide additional information that will help to have a better understanding of nucleation and crystallization, which is so crucial for obtaining glass-ceramics. We hope this comprehensive review will serve as a useful guide to students and researchers to clarify the short-range structure of oxide glasses to unveil the intricate details of different phenomena.
